# A comprehensive review on the use of algal-bacterial systems for wastewater treatment with emphasis on nutrient and micropollutant removal

**DOI:** 10.1080/21655979.2022.2056823

**Published:** 2022-04-20

**Authors:** Raj Kumar Oruganti, Keerthi Katam, Pau Loke Show, Venkataramana Gadhamshetty, Venkata Krishna Kumar Upadhyayula, Debraj Bhattacharyya

**Affiliations:** a Department of Civil Engineering, Indian Institute of Technology Hyderabad, Kandi, Sangareddy, India; b Department of Civil Engineering, École Centrale School of Engineering, Mahindra University, India; c Department of Chemical and Environmental Engineering, Faculty of Science and Engineering, University of Nottingham, Malaysia; d Civil and Environmental Engineering, South Dakota School of Mines and Technology, Rapid, South Dakota, USA; e Department of Chemistry, Umeå University, Sweden

**Keywords:** Algal-bacterial, micropollutant, quorum sensing, resource recovery, wastewater treatment

## Abstract

The scarcity of water resources and environmental pollution have highlighted the need for sustainable wastewater treatment. Existing conventional treatment systems are energy-intensive and not always able to meet stringent disposal standards. Recently, algal-bacterial systems have emerged as environmentally friendly sustainable processes for wastewater treatment and resource recovery. The algal-bacterial systems work on the principle of the symbiotic relationship between algae and bacteria. This paper comprehensively discusses the most recent studies on algal-bacterial systems for wastewater treatment, factors affecting the treatment, and aspects of resource recovery from the biomass. The algal-bacterial interaction includes cell-to-cell communication, substrate exchange, and horizontal gene transfer. The quorum sensing (QS) molecules and their effects on algal–bacterial interactions are briefly discussed. The effect of the factors such as pH, temperature, C/N/P ratio, light intensity, and external aeration on the algal-bacterial systems have been discussed. An overview of the modeling aspects of algal-bacterial systems has been provided. The algal-bacterial systems have the potential for removing micropollutants because of the diverse possible interactions between algae-bacteria. The removal mechanisms of micropollutants – sorption, biodegradation, and photodegradation, have been reviewed. The harvesting methods and resource recovery aspects have been presented. The major challenges associated with algal-bacterial systems for real scale implementation and future perspectives have been discussed. Integrating wastewater treatment with the algal biorefinery concept reduces the overall waste component in a wastewater treatment system by converting the biomass into a useful product, resulting in a sustainable system that contributes to the circular bioeconomy.

## Graphical Abstract

**Figure:**
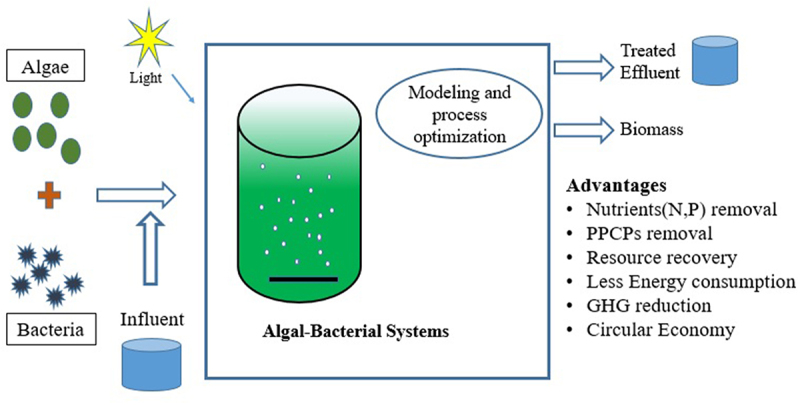

## 1. Introduction

Water is a necessary component of life on earth, and it covers almost 71% of the earth<apos;>s surface area. Recently, the scarcity of water has become a major threat around the world due to the over-exploitation of natural water resources. The natural water environment is continuously getting polluted due to industrialization and urbanization [1]. The quantity of wastewater generated is increasing because of population growth, industrialization, and urbanization. As per World Population Prospects 2019, the world<apos;>s population is expected to grow to around 8.5 billion in 2030, by 2050 to 9.7 billion, and near 11 billion in 2100 [2]. This continued increase in the population poses challenges for sustainable development. With the increasing population, changing socio-economic conditions, and variable consumption patterns, the quantity and the quality of wastewater are changing very fast, thereby imposing new challenges on existing wastewater treatment systems. According to the United Nation<apos;>s World Water Development Report 2017, around 80% of the total wastewater generated worldwide is disposed of without any proper treatment [3]. The quality of wastewater is changing due to the introduction of new chemical products for industrial, agricultural, and domestic consumption, leading to the occurrence of micropollutants or emerging contaminants. These emerging contaminants belong to the category of pharmaceuticals, personal care products, insecticides, surfactants, pesticides, plasticizers, and flame retardants [4]. The ecotoxicological effects of these emerging contaminants are still not very well known. Most of the conventional wastewater treatment plants in developing nations are neither monitoring nor are designed for removing these micropollutants, leading to their presence in natural water bodies [5]. Moreover, with the increasing restrictions on effluent disposal and due to the presence of a growing number of emerging micropollutants, which affect the performance of wastewater treatment plants, the plant operators are finding it difficult to meet the discharge criteria [6].

Several pharmaceutically active compounds (PACs) and heavy metals are present in wastewater streams. PACs have been greatly used to prevent and treat diseases affecting humans and animals in the fields of disinfection, aquaculture husbandry, disease diagnosis, epidemic prevention, disease treatment, and animal cultivation [7,8]. PACs have a complex and stable structure, low volatility, varying hydrophobicities among compounds, and their occurrence at trace levels, making their removal difficult using most of the available conventional treatment processes [9]. PACs (antibiotics) presence helps in the development of antibacterial resistance genes and thus are responsible in emergence of super-bacteria which has been considered as one of the emerging concern from the past few decades [8–12]. For example, diclofenac is said to cause antiovulatory effects on aquatic vertebrates, while the presence of ciprofloxacin may affect pathway of photosynthesis in higher plants which result in morphological abnormalities or growth inhibition [9]. Recently studies have shown that PACs exposure among humans has resulted in reproductive abnormalities, cognitive impairment, and miscarriages [10]. As PACs are designed to be biologically active even at lower levels, and thus their presence in aquatic environment can be harmful to both targeted and non-targeted aquatic as well as terrestrial [7,9,11,13]. The ubiquitous bioaccumulation nature of heavy metals, even at the trace level, and the persistent concentration increase in the environmental components has resulted in their possible uptake through food chain, atmosphere, and groundwater [14–17]. Studies have reported that long-term exposure to heavy metals in concentrations beyond the safe limits results in various health-related issues and can also cause death among some living organisms [14–16,18,19]. Heavy metals are genotoxic and carcinogenic and are reported to cause kidney and liver problems to among humans and aquatic systems [16,20]. Hence, the removal of these PACs and heavy metals from wastewater streams is necessary.

Apart from micropollutants, emphasis has also been given to nutrient removal in order to prevent adverse effects on receiving water bodies. Conventional biological nutrient removal processes involve several combinations of anaerobic, nitrification, and denitrification units. These systems require several reaction tanks and internal recycling leading to high operational costs, energy input, and process complexity. Due to the associated process complexities, these systems require trained personnel for the plant operation [21]. Conventional activated sludge processes primarily remove carbon from wastewater with lesser emphasis on nitrogen and phosphorus. The disposal of nutrient-rich effluent from the wastewater treatment plants leads to the eutrophication of water bodies [22]. Eutrophication has several ecological impacts, such as biodiversity reduction, water toxicity, and decreased lifespan of water bodies [23]. Previously, agriculture used to be considered as the major source for nutrient loading; but recently, wastewater from rapidly growing urban agglomerations in developing countries are emerging as the major contributing source of the same [24].

Wastewater treatment plants have environmental implications due to the greenhouse gas (GHG) emissions during the wastewater collection, treatment, and disposal. Existing conventional wastewater treatment systems, such as the activated sludge process, consume a lot of energy and have high operational costs. In some cases, 60% of the operating cost can be due to the aeration system only [25]. GHGs such as methane (CH_4_), carbon dioxide (CO_2_), and nitrous oxide (NO_2_) can be emitted from activated sludge processes directly or indirectly [26]. Indirect CO_2_ emissions contributing to the GHGs come from the facility<apos;>s energy usage, such as power supply used for aeration and pumping. As per US EPA<apos;>s report, wastewater treatment plants contribute around 5% of the global non-CO_2_ GHG emissions [27].

Several researchers have highlighted microalgae-bacteria association as potential consortia for low-cost wastewater treatment, particularly for nutrient removal, while simultaneously producing biomass that can be used for resource recovery [25,28–32]. Although the application of microalgae for wastewater treatment dates back to the 1960s, there has been an exponential increase in the research related to the topic in the last decade. This can be attributed to the increased awareness regarding climate change around the globe, carbon footprint and life cycle assessment of man-made systems, and search for sustainable practices.

Although several researchers have reviewed the prospects of wastewater treatment using algal-bacterial systems [29–31], a comprehensive review combining different aspects of algal-bacterial systems was not available. This review comprehensively discusses the algal-bacterial systems for wastewater by covering the nutrient removal, types of reactors, quorum sensing, and aspects of modeling along with resource recovery and future prospects. This paper aims to present the utilization of algal-bacterial systems for wastewater treatment based on the latest literature available. Section 2 discusses microalgal usage for wastewater treatment. The algal-bacterial symbiosis role in wastewater treatment and quorum sensing interaction between algae and bacteria has been presented in section 3 with schematic representations. Section 4 discusses the various types of available algal-bacterial reactor configurations, design parameters, and treatment capacities. The micropollutant removal mechanisms such as sorption, volatilization, biodegradation, and photodegradation have been discussed in detail with schematic illustration in section 5. Section 6 and section 7 presents the modeling aspects of algal-bacterial systems and aspects of biomass harvesting, respectively. The resource recovery aspects of algal-bacterial systems were discussed in section 8. Finally, section 9 presents the future prospects and challenges of using microalgal-bacterial systems for wastewater treatment.

## 2. Microalgae for wastewater treatment

Microalgae are unicellular eukaryotic microorganisms present in both fresh and marine water bodies. Microalgae are photosynthetic microorganisms that grow, produce oxygen, and biomass by utilizing sunlight, carbon from CO_2_, and inorganic nutrients. Recently, microalgal biomass has been extensively used for the biofuel synthesis, extraction of chemicals, and other bioproducts [33–36]. The microalgae remove nutrients through cellular uptake, and the produced biomass can be used for resource recovery [37]. The major benefit of using microalgae for wastewater treatment is the different multiple pollutant removal mechanisms. The pollutants in wastewater can be removed in an algal-bacterial system through several processes such as assimilation (uptake of nitrogen and phosphorus), stripping (ammonia removal at high pH), nitrification-denitrification, oxidation of organic carbon to carbon dioxide, and adsorption (heavy metals removal), phosphorus precipitation and pathogen removal due to pH fluctuations [1]. In conventional biological-nutrient removal processes, nitrogen removal is attained through sequential nitrification (aerobic) followed by denitrification (anoxic). Removal of phosphorus through the biological pathway requires a pre-anaerobic step. Therefore, the conventional biological nutrient removal process requires several reactors, which increases the complexity of the operations. Wastewater treatment using microalgae has been getting attention in recent years due to the latter<apos;>s high nutrient uptake ability [22,38,39]. Microalgae, through photosynthesis, can also increase the dissolved oxygen in wastewater. Other advantages include the generation of nutrient-rich algal biomass that can be utilized as animal feed and can be converted into fertilizers and biofuel. The most commonly used approach for wastewater treatment by microalgae is by using High Rate Algal Ponds (HRAP) or raceway ponds and Photobioreactors (PBRs). Several studies have reported that high nitrogen removal, 80–100%, can be achieved using HRAPs and photobioreactors [36,40–42]. However, even though high removal efficiencies can be achieved, microalgal wastewater treatment has some limitations. One of the main drawbacks of using HRAPs is that it requires a larger area; therefore, it may not be a feasible option of treatment everywhere [21,43]. Another drawback of the microalgae system is the poor settleability of the biomass. Suspended microalgae in effluent hinder achieving the TSS disposal standards [44].

## 3. Microalgae-bacterial systems for wastewater treatment

### 3.1. Algae-bacterial symbiosis and interactions

**Figure 1.**
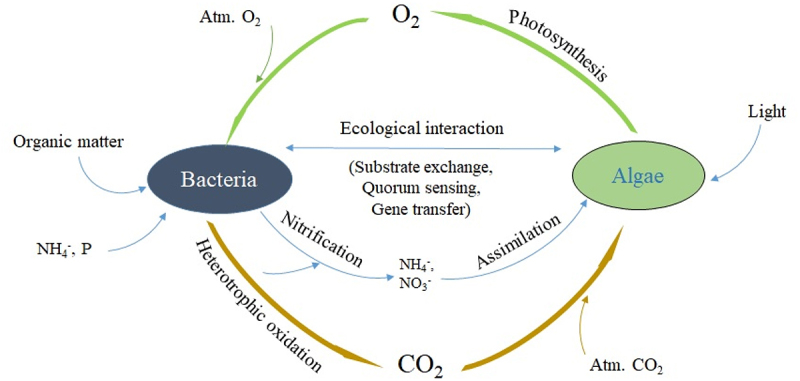
Schematic representation of algal-bacterial symbiosis.

For the microalgal-bacterial system to be competitive with the existing conventional processes like the activated sludge process, the design and operation of these systems should be such that faster removal rates can be achieved with lesser footprint and operational cost. Due to the simple operation, the robustness of the system, and higher removal efficiencies, algal-bacterial systems have been studied extensively in recent years [1,35,36,45–47]. It is well reported that algal-bacterial symbiosis occurs in waste stabilization ponds, oxidation ponds, and high-rate algal ponds [48]. The algal-bacterial symbiosis was found by Oswald and co-workers in oxidation ponds treating the wastewater. The schematic representation of the symbiotic relationship between algae and bacteria is shown in Figure 1. Microalgae utilize CO_2_ for photosynthesis, assimilate nutrients, and release oxygen into the effluent stream. The oxygen released by microalgae can be used for the metabolism by heterotrophic microorganisms (bacteria) for oxidizing organic matter and ammonia. Also, inorganic carbon, nitrogen, and phosphorus released during bacterial metabolism can be utilized by microalgae [47]. The algal-bacterial symbiotic relationship was proven to be enhancing the removal efficiencies [49]. Along with nutrient removal, the algal-bacterial consortium is also capable of removing micropollutants, heavy metals, pharmaceuticals, and personal care products [4,5,50,51]. Due to the symbiotic exchange of carbon dioxide (CO_2_) and oxygen (O_2_) between algae and bacteria, the microalgae-produced in-situ photosynthetic oxygen can substantially reduce the expenses and greenhouse gases related to the conventional mechanical aeration in activated sludge systems [52].

Nutrient removal in algal-bacteria consortia is superior in comparison to algal and conventional systems due to multiple pathways available via algal-bacterial symbiotic relation. Nitrogen removal in algal-bacterial systems occurs through several pathways. Nitrification-denitrification is also an important pathway for removal of nitrogen in addition to ammonium stripping due to high pH > 9 and assimilation by the biomass. The oxygen produced by the microalgae helps the nitrifying bacteria [53]. The anoxic zones present in the reactors help in the denitrification process [54]. Phosphorus is also a major essential nutrient for algal growth. Phosphorus can be removed by either chemical or biological processes. The chemical mechanism through which the phosphorus is removed is precipitation. At higher pH levels, usually around 9, the phosphorus will get precipitated. In the biological phosphorus removal mechanism, phosphorus gets assimilated into biomass through phosphorylation [23]. Though having several advantages, the competitive interaction and inhibitive mechanisms that exist in algal-bacterial systems are still not well known. Table 1 summarizes the algal-bacterial consortia used for wastewater treatment and removal efficiencies of carbon, nitrogen, and phosphorus.

**Table 1.** Different algae-bacterial consortia used for wastewater treatment

Culture	Type of wastewater	Mode Vol(L)	time H or d	C R(%)	N R(%)	P R(%)	Reference
*Chlorella vulgaris &* *Microcystis aeruginosa*	Synthetic wastewater	Batch 1 L	7 d	86.55 (COD)	88.95 (TN)	80.28 (TP)	[38]
Mixed algal – bacterial culture	MWW	Batch 14 L	14 d	91.2–96.2 (COD)	41.7–91.0 (TN)	64.0–93.7$\left({PO}_{4}^{3-}-P\right)$	[49]
*Chlorella vulgaris* & Activated sludge	Synthetic wastewater	Batch 30 L	2.7–4 d	78–86 (TOC)	33–66 (TN)	-	[256]
Mixed algal bacterial culture	MWW	Batch 14 L	8 d	95–98 (COD)	77–98 (TKN)	55–73$\left({PO}_{4}^{3-}-P\right)$	[56]
Mixed algal bacterial culture	Synthetic wastewater	Batch 1.4 L	12 h	96.7 (COD)	70.5 (TN)	89.9$\left({PO}_{4}^{3-}-P\right)$	[257]
Mixed algal bacterial culture	Synthetic wastewater	Semi batch 0.5 L	2 d	78–86 (COD)	97–99.9 (Nitrate)	94–99$\left({PO}_{4}^{3-}-P\right)$	[258]
*Chlorella and Scenedesmus*	Synthetic wastewater	Batch 2.0 L	2–5 d	-	36–66 (TN)	17.2–35.9$\left({PO}_{4}^{3-}-P\right)$	[66]
Mixed algal bacterial culture	Synthetic wastewater	Batch 4.0 L	12 h	80 (COD)	65.8 (TN)	89.3 (TP)	[127]
Mixed algal-bacterial culture	Synthetic wastewater	Batch 3.0 L	15 h	80–86 (COD)	30–50 (TN)	60–75 (TP)	[54]
Mixed algal bacterial culture	Domestic wastewater	Semi cont. 0.5 L	2–10 d	83–88 (TOC)	50–70 (TN)	80–93$\left({PO}_{4}^{3-}-P\right)$	[226]
Mixed algal bacterial culture	Primary treated MWW	Conti. 31 L	10 d	90 (TOC)	70 (TN)	85$\left({PO}_{4}^{3-}-P\right)$	[259]
Mixed algal bacterial culture	DWW	Batch 8000 L	4 d	-	92–97$\left({NH}_{4}^{+}-N\right)$	70–73$\left({PO}_{4}^{3-}-P\right)$	[41]

R (%)- Removal Efficiency; MWW-Municipal wastewater; DWW-Domestic wastewater; COD-Chemical oxygen demand; TOC-Total organic carbon; TN-Total Nitrogen; ${PO}_{4}^{3-}-P$ – Phosphate; TP-Total Phosphorus; ${NH}_{4}^{+}-N$-Ammonia Nitrogen; TKN-Total Kjeldahl Nitrogen

Nutrient uptake and consumption by microalgae for their growth can significantly reduce the nutrient levels in wastewater, thereby enhancing effluent wastewater quality [55]. For instance, Su et al. [56] reported biomass accumulation as the major nutrient removal mechanism, accounting for around 62 % and 45 % of the total inlet phosphorus and nitrogen while treating municipal wastewater with algal-bacterial culture. *Chlorella pyrenoidosa* cultivated in an open pond treating domestic wastewater showed NH_4_-N removal of 95 % and TP removal of 81% [57]. García et al. [58] reported that biogas scrubbing enhanced the TN removal from 31 to 81% and P-PO_4_^3-^ removal from 59 to 64%. This increase in the nutrient removal rate was attributed to the high algal growth rate facilitating high nutrient assimilation. The inoculum ratio of algal biomass to bacterial biomass can influence the treatment efficiency. Amini et al. [59] evaluated the effect of algal to activated sludge inoculum ratio (5:1, 1:1, and 1:5) on the performance of semi continuous photobioreactors treating domestic wastewater. They have observed that the inoculum ratio of 5:1 showed maximum ammonium and phosphorus removal efficiency. An increase in nitrogen removal can be achieved with an increment in the inoculum ratio of algae to bacteria [60].

The algal-bacterial association is not just limited to the exchange of carbon dioxide and oxygen, but it covers a wide range of other possible interactions. The algal-bacterial interaction may exhibit mutualism, commensalism, and parasitism [61]. Mutualism is a process of ecological interaction in which both species are benefitted from each other. For example, several researchers have reported that the bacteria supplied vitamin B_12_ to the microalgae, and in exchange, microalgae supplied the fixed carbon to the bacteria [62–64]. In another study, Kim and co-workers [65] showed that *Rhizobium sp*., when co-cultured with *Chlorella Vulgaris*, promoted the algal cell count by 72% due to the mutualistic relationship. Several studies have stated that the formation of granules or aggregates improves the biomass settling properties in algal-bacterial cultures [44,66,67]. The extracellular polymeric substance formation due to the mutualism has been reported as the major reason for microalgal-bacterial flocs generation, which benefitted the downstream processing [68]. The algal-bacterial associative interaction can compensate for the micronutrient deficiency, which is a basic requirement for growth. To overcome the limitation of key micronutrients, the bacteria and microalgae produce siderophores, which bind to the needed element and increase its solubility. For example, Amin and co-workers [69] have reported that the bacteria promoted algal iron uptake by facilitating the photochemical redox cycling of siderophore chelate compound Fe-vibroferrin. In return, microalgae released organic compounds such as amino acids, sugars, and lipids that are used for bacterial growth promotion in a mutualistic relationship. In a commensalism type of relationship, one of the species gets benefits while the other neither receive any discernable benefit nor get harmed. A study done by Villa and co-workers [70] showed that the *Azotobacter vinelandii* could fix the nitrogen under reduced carbon availability by producing a variety of siderophores. The microalgal species *Scenedesmus sp*. BA032 and *Neochloris oleoabundans* were able to utilize the siderophore azotobactin as a nitrogen source [70]. Parasitism is a way of symbiotic relationship in which one species, called a parasite, lives on another species, thereby causing harm to the latter. Most of the bacteria negatively affect the microalgae and are extensively used to control the algal blooms [71]. The lysis of algal cells by the action of cellulases and other enzymes leads to the bacterial utilization of intracellular compounds [72]. Moreover, another way of parasitism exists, in which the competition for available nutrients results in a slower growth rate of a particular species and eventually outcompeting their existence after several generations [61]. For example, Zhang et al. [73] reported that the algae species *Chlorella pyrenoidosa* might hinder the growth of the bacteria under high carbon concentrations in the medium. However, these interactions exist in a continuum, and the lines that delineate mutualism, commensalism, and parasitism are not clear [74]. The plasticity of these interactions from mutualism to parasitism via commensalism is strongly dependent on the environmental conditions [72]. For example, Cabrerizo and co-workers [75] demonstrated that the incident solar radiation and nutrient pulses are able to regulate ecological functioning by changing from photoautotrophic to mixotrophic conditions. Another study done by González-Olalla et al. [76] shows that depending on temperature and nutrient availability, the algal-bacterial interaction shifted from bacterivory regulated by algae to commensalism.

#### 3.1.1 Quorum sensing molecules and their effect on algal-bacterial population dynamics

**Figure 2.**
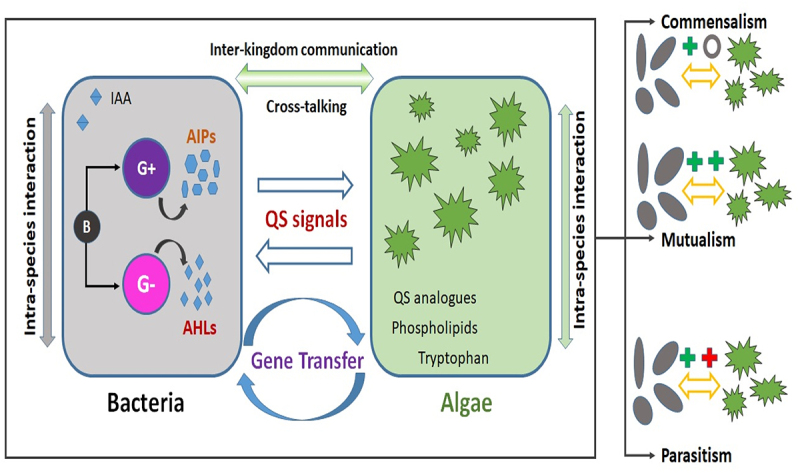
Schematic illustration of quorum sensing molecules interaction in algal-bacterial systems. (G+ – gram positive bacteria; G- – gram negative bacteria; AIPs – auto inducing peptides; AHLs – Acyl-homoserine-lactones).

The interaction between algae and bacteria covers the substrate exchange, cell to cell communication, and horizontal gene transfer. The cell to cell communication process between the species present in an ecological system is known as quorum sensing (QS). QS is a population-dependent interaction mechanism in bacterial cells facilitated by exchanging small signaling molecules, which helps in coordinating gene expression and performing ecological functions [28]. These interactions are caused by signaling molecules such as auto-inducers (AI-2), N-acyl-homoserine lactones (AHLs), indole-3-acetic acid (IAA), and auto-inducing peptides (AIP). Gram-negative bacteria usually secrete tiny sensing molecules such as AHLs, while gram-positive bacteria secret AIPs as sensing molecules [77]. The schematic illustration of the quorum sensing and algal-bacterial interactions has been shown in Figure 2. The complex interactions of these signaling molecules are expected to occur in the phycosphere, a diffusive boundary layer region surrounding the algal cell in which the algal exudates influence co-occurring organisms [78]. Microalgae can provide nutrients for bacteria and stimulate their proliferation by secreting substances that also can enhance biofilm formation [79]. Microalgae have the capability for sensing the bacterial quorum signals and respond accordingly. For example, Zhou and co-workers [80] investigated the effect of signaling molecule AHLs extracted from bacterial activated sludge on microalgae *Chlorophyta sp*. The findings of the study showed that with the addition of AHLs, the algae formed self-aggregated flocs of 200 μm while enhancing the settling efficiency from 8% to 41%. This was attributed to the secretion of aromatic protein by algae in response to the bacterial AHLs, which was supported by transcriptomic analysis [80]. In support of this, Zhang and co-workers [81] provided evidence on the role of bacterial AHLs on the algal-bacterial granular sludge development. In another study, Amin et al. [82] reported that the bacterial species *Sulfitobacter* promoted the cell division in diatoms by secreting indole-3-acetic acid. Bacterial movement and biofilm development are controlled by QS. For instance, Fei et al. [83] have shown that the QS influences the colonization of diatom surfaces by the bacteria. The study evaluated the association of three bacterial symbionts – *Phaeobacter sp. F10, Alteromonas macleodii F12, and Sulfitobacter pseudonitzschiae F5* with the diatom *Asterionellopsis glacialis*. The results showed that even though all three bacteria have the required genes necessary for motility and attachment, only two symbionts were able to synthesize QS signals. Zhang et al. [81] have explored the role of AHLs compounds in algal bacterial granular systems. They have reported that at a lower light intensity, higher production of AHLs mainly C6-HSL and 3OC8-HSL, contributed to the EPS production and biofilm formation. Das et al. [84] have reported that the addition of quorum sensing molecules recovered from anaerobic sludge to the microalgal culture *Chlorella Sorikiniana* increased the algal productivity and lipid content by 2.25 and 1.8 times respectively. The bacteria QS molecules were identified as bacterial siderophores, autoinducing oligopeptides, N-Hexanoyl-L-homoserine lactone, and N-3-oxohexanocyl-L-homoserine lactone. In the same study, it was reported that the algal cells secreted QS inhibitory molecules such as β cyclodextrin, dimethyl sulphohonio propionate, 5-4-5-bromomethylene-3-butyl-2-5 H-furanone, and halogenated furanones for the bacterial toxins inactivation. Microalgae responses to the environmental pressures, such as bacterial competition, are self-protective responses. For example, Zhang et al. [85] reported that when the QS molecules, extracted from activated sludge, were added to *Chlorophyta* sp. culture, the lipid productivity increased by 84 %. This increase in lipid productivity was ascribed to the addition of QS molecules that stimulated Acetyl-CoA enzyme production which is the primary compound in fatty acid synthesis. To confirm this, in the same study, they have added synthetic QS molecule C6-HSL (N-hexanoyl-L-Homoserine lactone) to promote the lipid synthesis. The biomass productivity and lipid productivity stimulated by synthetic C6-HSL were identical to the QS molecules extracted from the activated sludge. Ji et al. [38] have evaluated the algal-bacterial symbiotic relationship between *Chlorella sp*., and *Bacillus licheniformis* by analyzing the quorum sensing molecules which auto-induced peptides (AIP) and bis (3’-5’) diguanylic acid (c-di-GMP).

Algae and bacteria can interact between their cross kingdoms and is a new area of research [86]. IAA is a widely known cross-talk signal molecule. This endogenous phytohormone found in plants and algae can be produced and released by a variety of bacterial species. For instance, Dao et al. [87] have reported that bacterial strains isolated from *Scendesemus* sp. promoted the algal growth by producing the IAA. In the same study, it was reported that microalgae also secreted signal substances and IAA synthetic substance (tryptophan). In a further study, Peng et al. [88] showed that bacterial species *Azospirillum Brasilense* promoted the growth of algal species *Chlorella sorokiniana* and *Auxenochlorella protothecoides* by producing IAA. However, the effect of IAA or *A. brasilense* was species-dependent showing higher growth promotion in *C. sorokiniana* than that of *A.protothecoides*. High IAA secreting bacterial species such as *Rhizobium* and *Agrobacterium* could enhance the microalgal growth by 65–80% compared to the control sample [89]. AHLs are the QS molecules produced by gram-negative bacteria, which promote extracellular polymer substance (EPS) production, microbial attachment, and biofilm formation [90]. The activity of quorum sensing molecules plays a significant role in the formation of biofilm. Liu et al. [90] has reported that high levels of AHLs were observed in younger biofilms than mature biofilms. The low AHLs levels were attributed to an increase in the proportion of quorum quenching (QQ) bacteria in mature biofilms. For instance, Güneş and Taşkan [91] used the QQ strategy for controlling biofouling in membrane photobioreactor using *Rhodococcus* sp. BH4 as QQ bacterial species. They have reported a reduction in EPS production and cake formation on biofilm, leading to an overall decrease in transmembrane pressure by 37% compared to control. There are still more knowledge gaps in the understanding of these algal-bacterial interactions. The exploration of sensing mechanisms between algae and bacteria is much needed and helps in identifying the suitable strategies for real scale systems.

#### 3.1.2 Horizontal gene transfer

Horizontal gene transfer (HGT) is a crucial factor in the evolutionary process, in which the genetic material is exchanged horizontally rather than a parent to offspring via vertical transmission. HGT is ubiquitous in microalgae due to a long history of the co-evolution process. This HGT makes the eukaryotic microorganisms functionally diversified and capable to survive in extreme environments. For example, the phylogenetic analysis of *Galdieria sulphuraria*, an extremophilic red algae species that survives in hot, toxic, and acidic environments, revealed that this species acquired exceptional metabolic capabilities to grow either heterotrophically or photoautotrophically, by utilizing more than fifty carbon sources, from at least 75 genes of different bacteria and archaea by HGT [92]. Marchetti and co-workers [93] showed that pennate diatoms, *Fragilariopsis* and *Pseudo-nitzschia*, have acquired the capability to synthesize iron-concentrating protein, ferritin, via lateral gene transfer. Most of the functionally significant genes are laterally transferred to eukaryotes from bacteria because of the vast metabolic diversity of the bacteria [94]. HGT transfer plays an important role in adaptive advantages to environmental stresses.

### 3.2 Algae-bacterial treatment systems

These are of two types: suspended growth and attached growth systems.

#### 3.2.1. Suspended growth systems

Algae-bacterial consortia in suspension have been used for wastewater treatment for several decades and include open and closed bioreactors. Open bioreactors are pond systems that can be either natural or artificial.

##### High rate algal pond (HRAP)

**Figure 3.**
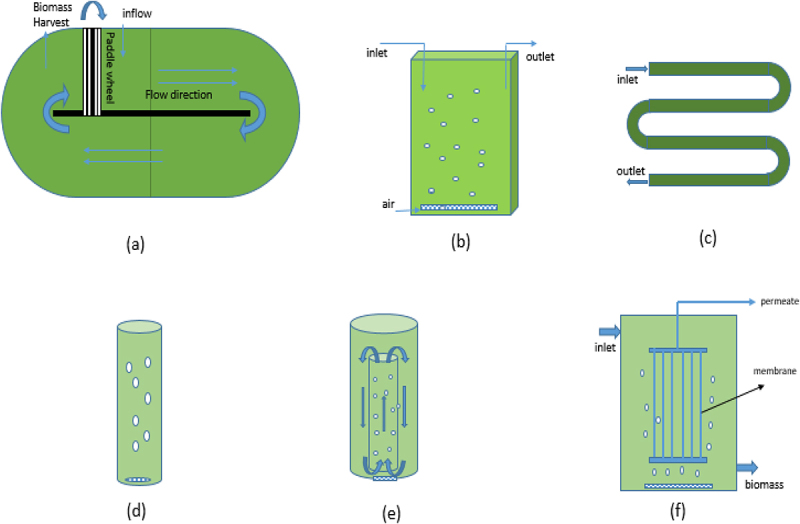
Schematic representation of algal-bacterial systems (a) high rate algal pond (b) flat-plate PBR (c) tubular PBR (d) bubble column PBR (e) internal-looping column PBR (f) membrane PBR.

HRAPs are the most extensively utilized algal-bacterial systems for wastewater treatment [95–98]. HRAPs have the ability to treat wastewater with low consumption of energy compared to traditional wastewater treatment processes while at the same time producing valuable biomass for resource recovery [99]. HRAPs were first developed at the University of California for wastewater treatment using algal biomass [100,101]. HRAPs are low cost wastewater treatment techniques compared to conventional activated sludge treatment techniques [102]. The schematic representation of the HRAP has been shown in Figure 3(a). The HRAP design includes a raceway-style pond with shallow water depths, typically 0.2–1.0 m. The gentle mixing is provided using a paddle wheel, giving a horizontal flow velocity of 0.15–0.3 m.s^−1^. This allows the proliferation of microalgae which results in nutrient removal due to the latter<apos;>s assimilation into biomass [103]. The algal photosynthesis results in high dissolved oxygen, which facilitates the aerobic microbial degradation of organic matter. Because mechanical aeration is not required, these systems consume far less energy than the activated sludge process (0.02–1 kWh/m^3^ of water) [104]. The design parameters, algal productivity, and nutrient removal efficiency of HRAPs have been summarized in Table 2. The depth of the HRAP is one of the decisive factors which influences biomass productivity. Arbib et al. [105] has shown that a pond with a 0.3 m depth yielded higher biomass productivity than one with a depth of 0.15 m. This increase in the productivity with the depth is because of the increase in photosynthetic efficiency of the algal cells under light-limiting conditions [101]. In another similar study, Sutherland et al. [106] investigated the performance of the HRAPs operated at three different depths, viz. 200, 300, and 400 mm. They have also reported that overall areal microalgal productivity increased with an increase in depth. However, after a certain depth, the light intensity was so low that the photosynthetic activity ceased [101].

**Table 2.** Design parameters, nutrient removal efficiency and algal productivity of high rate algal ponds

Wastewater type	Volume (m^3^)	Length (m)	Width (m)	Depth (m)	Surface area(m^2^)	Microalgal Productivity gVSS/m^2^.d	HRT (day)	Removal %			Reference
								C	N	P	
MWW	0.47	-	-	0.3	1.5	20 ± 7	4.5	65 ± 23 (COD)	48 ± 16 (TN)	25 ± 52 $\left({PO}_{4}^{3-}-P\right)$	[260]
DWW	9.54	-	2.5	0.3	31.8	2.0–11.1	5–8	-	31–92$\left({NH}_{4}^{+}-N\right)$	32–76$\left({PO}_{4}^{3-}-P\right)$	[261]
Secondary Effluent	0.533	2.525	0.750	0.3	1.93	26.2 ± 1.2	5	-	-	-	[105]
DWW	8	-	-	0.3	31.8	9–16.7	4–8	95 (COD)	-	-	[262]
UWW	22	-	-	0.3	73.4	30–65	6–2.5	-	-	-	[263]
Primary settled wastewater	0.43	1.8	0.6	0.25	2.0826	-	2 4 6 8	-	64 81.8 85.38 81.81 (TN)	51.1 62.26 77.81 75.78 (TP)	[98]
Primary settled wastewater	0.52	1.8	0.6	0.3	2.0826	-	2 4 6 8	-	56.39 84.5 89.1 91.7 (TN)	41.92 66.1 82.81 76.85 (TP)	[98]
SHW	0.075	1.25	0.6	0.18	0.43	12.7	10–15	84–91 (COD)	70–80$\left({NH}_{4}^{+}-N\right)$	57–90$\left({PO}_{4}^{3-}-P\right)$	[264]
MWW	0.06	-	-	0.3		0.5 ± 0.03	2	85.44 ± 5.10 (COD)	92.74 ± 5.82 (TN)	82.85 ± 8.63 (TP)	[265]
DWW	0.180	1.7	0.82	0.15	1.33	5	10	77 ± 9 (COD)	83 ± 10 (TKN)	94 ± 6 (TP)	[266]
DWW	-	30	5	0.32	200	-	5	91.76 (BOD)	-	-	[267]
MWW	4375	-	-	0.35	12,500	9.7	5.5–9	-	47–79$\left({NH}_{4}^{+}-N\right)$	20–49$\left({PO}_{4}^{3-}-P\right)$	[268]

MWW-Municipal Wastewater; DWW-Domestic wastewater; SHW-Slaughterhouse wastewater; COD-Chemical oxygen demand; TN-Total Nitrogen; ${PO}_{4}^{3-}-P$ – Phosphate; TP-Total Phosphorus; ${NH}_{4}^{+}-N$-Ammonia Nitrogen; TKN-Total Kjeldahl Nitrogen; BOD-Biochemical oxygen demand; ‘-‘ – not available

For increasing biomass productivity and nutrient removal, several operating strategies have to be adopted. Recently, Sutherland et al. [107] have evaluated the performance of HRAPs operated in series at HRT of 4 days versus reactors in parallel conditions operated at an HRT of 8 days. It was observed that microalgal productivity and nutrient removal were more in the series type than the parallel one. The microalgal productivity in full scale HRAPs is often lesser than the pilot scale HRAPs regardless of environmental conditions. In another study, Sutherland et al. [108] reported the effect of the size of the HRAPs on the microalgal productivity by comparing the performance of three different HRAPs having areas- 5 m^2^ (mesocosm), 330 m^2^ (pilot scale), and 1 hectare (full scale). They reported that the microalgal productivity was more in pilot scale compared to the other two systems. This increase in the microalgal productivity was attributed to higher mixing frequency and increased photosynthetic capability under light limiting conditions in the pilot scale system. As the size of the HRAP increases, the intensity of vertical mixing decreases, and the laminar regime induces dead zones in long channels leading to the sedimentation of the algal cells at the bottom of the pond [109]. It was also reported that the increase in vertical mixing favored the medium dark/light cycles under low light resulting in higher photosynthesis in smaller HRAPs. Microalga production and wastewater treatment in HRAPs can be increased by optimizing the light penetration by modifying pond depth or algal concentration and hydraulic retention time as per season [110]. However, the HRAPs require a large area compared to other treatment systems which is very difficult to provide nowadays.

One of the drawbacks of the HRAPs is the difficulty in separating biomass after treatment. In HRAPs, the biomass consists of microalgae and mixed bacterial populations forming spontaneous flocs (20–200 μm), which can be partially settled by gravity without any chemical addition [44]. Gutiérrez et al. [95] studied the effect of biomass recycling (2–10% on dry weight) on the harvesting efficiency of biomass. The results showed that recycling improved the harvesting efficiency to 95% with settling velocities greater than 1.6 m/h. Recycling of biomass induces changes in species diversity. It was shown that 10% (on dry basis) of biomass recycling reduced the *Chlorella sp*. dominance by increasing the rapidly settling species such as *Stigeoclonium sp*. (which is only present in the recycled reactor) and diatoms from 0.7% to 7% [95].

##### Photobioreactor (PBR)

Photobioreactor (PBR)s provide controlled and suitable environmental conditions (light, nutrients, temperature, mixing, etc.) for algal growth [111]. In the open cultivation systems, maintaining monoculture strains is difficult as there is a chance of invasion by other species. Various types of PBR configurations are available such as flat-plate, column, tubular, airlift reactors, stirred tank., hybrid PBRs. The schematic representation of different types of photobioreactors has been shown in Figure 3(b-f). One of the earliest forms of the PBRs developed was Flat-plate PBR. Flat plate PBR consists of a flat thin surface media on which microalgae gets attached. The advantages of Flat plate PBRs include high illumination surface to volume ratio and minimal mechanical requirements [112,113]. Yang et al. [114] cultivated *Scenedesmus Obliquus* and *Chlorella Vulgaris* on vertical flat plate PBR and obtained more than 99% nutrient removal at a retention time of 8 days while treating municipal wastewater. One of the limiting factors in flat-plate PBRs is light attenuation inside the reactor due to the biomass. To reduce the light shading effect Sun et al. [112] embedded hollow polymethyl methacrylate (PMMA) tubes as light guides which resulted in a 23.42 percent increase in biomass production compared to that of without PMMA tubes.

Column PBRs are configured in vertical column position. The air or CO_2_ is supplied in the form of bubbles for mixing and generating turbulence needed for adequate suspension. Column reactors have a defined circular fluid flow and efficient gas-liquid transfer [111]. The column<apos;>s diameter should not exceed 0.2 m to avoid light attenuation inside the reactor. The height of the column is restricted to 4 m concerning the structural reasons and mutual shading effect [115]. The agitation caused by the bubbles induces a little shear stress compared to mechanical mixing, which favors the granulation process [116]. Different configurations of column PBRs are available such as bubble column PBR, draft-tube airlift column PBR [117], split column airlift PBR [118], external-loop column airlift PBR [115]. A water-circulating column photobioreactor (WCC–PBR) developed by Yang et.al [119] showed an energy consumption of 21.1 % lower than airlift column PBR operating with only an air compressor. Various types of photobioreactors and their operating conditions, nutrient removal efficiencies, and biomass productivities have been summarized in Table 3.

**Table 3.** Different PBRs operation conditions and their biomass productivities

PBR configuration	Algal species	Light (μmoles/m^2^/s) and Photoperiod (light-dark cycle) h	Biomass productivity	HRT (day)	Removal %			Reference
					C	N	P	
Flat-plate	*Nannochloropsis salina*	150–600 12:12	0.2 g L^−1^d^−1^	4.7	-	-	-	[269]
Flat-plate	*Scenedesmus ovalternus*	1300 24–0	25.0 ± 0.5 g m^−2^d	-	-	-	-	[270]
Full scale tubular PBR	*Sphaeropleales*	Sunlight	-	2–5	85.86 ± 1.24 (COD)	-	-	[271]
Tubular PBR	*Chlorella protothecoides*	138 16–8	1.96 g L^−1^	10	78.03 (COD)	100 (TN)	100 (TP)	[272]
Air lift PBR	*Chlorella sorokiniana*	Sunlight	-		86.84 (COD)	100 (TN)	100 (TP)	[273]
Column PBR	Mixed algal-bacterial culture	121 ± 7.3 12–12	-	0.5	95.5–96.7 (COD)	60.4–70.5 (TN)	93.2–96.4$\left({PO}_{4}^{3-}-P\right)$	[257]
Bubble column PBR	*Chlorella sp. FC2*	1130 12–12	0.27–0.85 g L^−1^d^−1^	6–16	-	-	-	[274]
Osmotic membrane PBR	*Chlorella vulgaris*	46 24–0	2 g L^−1^	1–2	-	92–99$\left({NH}_{4}^{+}-N\right)$	100$\left({PO}_{4}^{3-}-P\right)$	[275]
Membrane PBR	*Chlorella vulgaris*	46 24–0	2 g L^−1^	1–2	-	84–97$\left({NH}_{4}^{+}-N\right)$	28–47$\left({PO}_{4}^{3-}-P\right)$	[275]
Membrane PBR	*Chlorella vulgaris*	101.5–112.3 24–0	27–49 g L^−1^d^−1^	1–6		36–92 (DIN)	77–95 (DIP)	[276]

COD-Chemical oxygen demand; TN-Total Nitrogen; ${PO}_{4}^{3-}-P$ – Phosphate; TP-Total Phosphorus; ${NH}_{4}^{+}-N$-Ammonia Nitrogen; DIN-Dissolved Inorganic Nitrogen; DIP-Dissolved Inorganic Phosphorus;‘-‘ – not available

#### 3.2.2 Attached growth systems

##### Algal turf scrubber (ATS)

ATS was created with the goal of promoting the natural wastewater treatment process. Algal Turf Scrubbing technology was developed by Adeay and his co-workers at Smithsonian Institution, Washington D.C in a way of promoting natural wastewater treatment systems [120,121]. An ATS consists of long inclined beds which support the formation of biofilm. The biofilm is composed of a mixed community containing filamentous microalgae, also called periphytons and epiphytic diatoms algae, along with aerobic bacteria and fungi. The wastewater is allowed to flow down the biofilm, while nutrients are removed by assimilation into the biomass. Predominant species observed were cyanobacteria (particularly *Oscillatoria sp*.,) and diatoms (*Nitzschia sp., Navicula sp., and Cyclotella sp*.) [121]. ATS technology overcomes many inherent problems associated with the growth of microalgae and the harvesting process. The benthic algae grown on the turf scrubber is scraped off once in a while into a container. In a way, increasing the ease of harvesting and reducing the overall biomass production cost [122]. Harvesting rates play a vital role in ATS technology. Siville and Boeing [123] have shown in a recent study that ATS was optimized by adapting the harvest rates between 7 to 14 days while simultaneously maximizing biomass production. In the same study, it was shown that nutrient removal does not get affected by harvest rate because of the trade-off between the biomass growth rate and frequency of harvest rate [123]. The hybrid system of ATS in series with the constructed wetlands showed a significant increase in the total nitrogen removal [124]. Different algal-bacterial biofilm reactors, their biomass productivity and nutrient removal capacities has been summarized in Table 4.

**Table 4.** Algal-bacterial biofilm reactors – operational conditions and removal efficiencies

Culture media	Reactor configuration	Species	Light(μmoles/m^2^/s) and Photoperiod(light-dark cycle) h	Biomass productivity (g m^−2^ d^−1^)	HRT Days	Removal %			Reference
						C	N	P	
UWW	Algal floway	*Nitzscia palea, Nitzschia umbanota, Nitzschia amphibia*	780–1147 12–12	34.83	-	-	-	-	[277]
SWW	Trickling filter	Mixed algal-bacterial culture	15 12-12	-	0.3–0.5	85 (TOC)	15 (TN)	49${PO}_{4}^{3-}-P)$	[126]
BG11 media	Algal turf scrubber	Benthic polyculture	32 watt 24	3.5	-	-	5–25$\left({NH}_{4}^{+}-N\right)$	31–70$\left({PO}_{4}^{3-}-P\right)$	[123]
Centrate wastewater	Algal turf scrubber	Mixed algal-bacterial culture	88 ± 16 16–8	-	10	91 ± 3 (TOC)	70 ± 8 (TN)	85 ± 9$\left({PO}_{4}^{3-}-P\right)$	[259]
Secondary wastewater	Algal turf scrubber	Mixed culture	6 watt 24	-	-	72 (COD)	70 (TN)	44$\left({PO}_{4}^{3-}-P\right)$	[124]
SWW	Biofilm carrier	*Scenedesmus sp*., *Chroococcus sp*., *Closterium sp*., diatoms, *Oscillatoria* *sp. and Chlorella sp*.	200 24	-	0.5	90 (COD)	90$\left({NH}_{4}^{+}-N\right)$	30$\left({PO}_{4}^{3-}-P\right)$	[125]
Secondary effluent	Algal turf scrubber	*Oscillatoria sp. Navicula sp., Nitzschia sp. Cyclotella sp*.	-	24	-	-	40 (TN)	50 (TP)	[121]

DWW-Domestic wastewater; SWW-Synthetic wastewater; UWW-Urban wastewater; COD-Chemical oxygen demand; TOC-Total organic carbon; TN-Total Nitrogen; ${PO}_{4}^{3-}-P$ – Phosphate; TP-Total Phosphorus; ${NH}_{4}^{+}-N$-Ammonia Nitrogen; ‘-‘- not available

##### Hybrid biofilm photobioreactors

Low hydraulic loading and high reactor footprints are restricting the wide application of algal-bacterial plane biofilm reactors. Recently, few studies have shown that by using three-dimensional media as a biofilm carrier, hydraulic retention times of less than a day could be achieved. For example, Gou et al. [125] reported that by using polyethylene three-dimensional biofilm carriers, satisfactory treatment performance could be obtained within 12 h of hydraulic retention time. Also, in a recent study, Katam and co-workers [126] used the polyurethane sponge cubes in an algal-bacterial trickling filter for treating domestic wastewater. In their study, it was reported the C, N, and P removal of 90%, 24%, and 37% was achieved within 8 h HRT. Tang et al. [127] used spherical carrier media supported on polymethyl methacrylate layer for algal-bacterial biofilm formation in a sequential batch reactor with 12 h HRT. They have reported that there was a significant increase in nitrogen and phosphorus removal efficiencies compared to sequential batch biofilm reactors. Choudhary et al. [128] have used nonwoven spun-bond fabric as the support for biofilm and achieved biomass productivity of 3.62 g/m^2^/d while treating domestic grey water.

##### Membrane aerated biofilm reactor (MABR)

Membrane aerated biofilm reactor (MABR) is a novel type of wastewater treatment system in which the hydrophobic membrane acts as a biofilm carrier and is used as bubble-free air diffuser. In a recent study, Zhang et al. [129] have shown that a significant increase in nitrogen removal efficiency was observed in a membrane aerated bacteria-algae biofilm reactor (MABAR) in comparison with MABR. The increase in nitrogen removal was attributed to the algal assimilation.

## 4. Physio-chemical factors affecting algal-bacterial systems

Many factors affect the interaction between the algae and bacteria, ranging from species-specific interactions, operating conditions such as hydraulic retention time and solids retention time, as well as environmental factors such as temperature, pH, light intensity, photoperiod (light-dark cycle), and mixing conditions [130,131]

### 4.1 pH & temperature

Every biological system is inherently dependent on the pH and temperature of the system. For most microalgal species, the ideal pH for growth is 8.2–8.7, although it can be buffered between 7 and 9 [111]. The pH of the system depends on the CO_2_ supplied due to the chemical equilibrium between H_2_CO_3_, HCO_3_^−^, and CO_3_^−2^ species. It is very common to notice that the consumption of CO_2_ by the algal biomass causes an increase in pH [132]. Also, sometimes the excessive supply of CO_2_ will drop the pH to the acidic region leading to algal cell lysis [133]. Therefore, the pH of the system should be controlled at the optimum range to favor algal growth.

Temperature, which directly influences the biochemical processes, is a crucial component in the growth of microalgae and bacteria. Each species has an optimum temperature range within which it will grow at the fastest rate. Outside the range, the biomass growth rate slows down and stops abruptly at one point. The temperature changes are inevitable due to diurnal cycles and seasonal variations. For most of the algal species, the most suitable temperature is 20–30°C. Increases in temperature within the optimum range have a positive influence on the photosynthesis process and cell division due to Calvin cycle related activities [134]. The temperature change affects the photosynthesis activity due to the complex kinetics of the ribulose-1,5-bisphosphate (Rubisco) enzyme. This particular enzyme is engaged in two pathways with the dual role of carboxylase and oxygenase activity. The Rubisco enzyme<apos;>s carboxylase activity increases with temperature up to a particular point. Above 30°C, the affinity of the enzyme towards CO_2_ reduces, thereby decreasing the photosynthetic activity [135]. Mayo [136] reported that at a temperature more than 40°C, *Chlorella vulgaris* species become less tolerant to acidic pH. Because at higher temperatures, the cytoplasm gets damaged, which leads to hydrogen ion penetration into chloroplasts. Lower temperatures affect the growth by altering the enzymatic activities of the cells [111,137]. In algal-bacterial systems, the microalgae coexist with heterotrophic bacteria, nitrifiers, and ammonium oxidizing bacteria (AOB) [138]. González-Camejo et al. [139] evaluated the ambient temperatures effect on the consortium of microalgae (*Chlorella sp*) and nitrifying bacteria. The performance is unaffected by temperature changes between 15 and 30°C; however, at temperatures above 30°C, microalgal activity is completely stopped. At the same time, an increase in the temperature increased the AOB leading to a competition for ammonia among the species. The lipid accumulation of the microalgae is also strongly influenced by temperature and it is dependent on the type of species. A temperature rise from 20 to 25°C increased the lipid accumulation in *N. oculata* sp. (from 7.90 to 14.92%), while a temperature increase from 25 to 30°C decreased the lipid content from 14.71 to 5.90% in *C. vulgaris* sp [140]. The response of the algal-bacterial strains to low and high temperatures is species-specific. Wu and co-workers [141] reported that at 25°C, they had observed maximum microalgal lipid content (32.9%), whereas maximum lipid productivity and biomass concentration were achieved at 30°C for *Monoraphidium sp. SB2* strain cultivated in a synthetic medium.

### 4.2. Light intensity

Microalgae, being phototrophic organisms, synthesize organic matter in the presence of light by assimilating nutrients and dissolved inorganic carbon. Several studies have shown that the light intensity and the light-dark cycle period are the major factors influencing the productivity of microalgae [35,142]. It is well documented that the changes in light intensity cause an immediate effect on organic removal rates, algal photosynthesis, and nitrification process by bacterial cells [143]. Lee et al. [142] investigated the effect of photoperiod conditions (12:12, 36:12, and 60:12 h dark-light cycles) on the removal of nutrients and biomass productivity. The result shows that the carbon removal was positively related to the length of the dark period and inversely with regard to nitrogen and phosphorus removal. The light intensity also influences the formation of settleable algae-bacterial granules. From a study conducted by Zhang et al. [81], it was observed that an algae-bacterial granular sludge, cultured at low intensity (142 ± 10 μmol m^−2^.s^−1^), has shown superior settling characteristics. The reason was attributed to the production of extracellular polymeric substances predominantly composed of tryptophan and aromatic proteins having much larger weight [81]. Light intensity also has an effect on lipid accumulation, oxygen production, and biological community structure [144]. Providing high light intensities will cause photo-inhibition in microalgae and nitrifying bacteria. It was reported by Vergara et al. [145] that providing high light intensity increases nitrite accumulation due to photo-inhibition of nitrite-oxidizing bacteria (NOB). Kang and co-workers [146] studied the effect of blue light with supplementary aeration on carbon and nitrogen removal efficiency. When algal biomass was weakly irradiated with blue light (500 μmol m^−2^s^−1^) and provided with supplementary aeration, the ammonia removal efficiency increased from 38.5% to 96.3%, and the algal growth also increased from 72.5 mg L^−1^ to 345.3 mg L^−1^. It was reported that the increase in the intensity of blue light led to the photo-inhibition of nitrite-oxidizing bacteria (NOB), whose c-type cytochrome is photo-bleachable at 408 nm [146]. However, higher biomass concentrations in the reactors obstruct the light penetration; therefore, nitrifying bacterial photo inhibition can be reduced significantly due to higher biomass concentrations [145]. Therefore, it can be understood that the light intensity and light/dark cycle are required to be optimized for the real-scale application of algae-bacterial systems for wastewater treatment.

### 4.3 Nutrients and C/N/P ratio

The microalgae biomass has a representative stoichiometric formula as C_106_H_181_O_45_N_16_P [147–149]. C/N/P ratio of algal biomass is approximately 50/8/1 on weight basis [150]. Most wastewaters do not have a similar ratio required for the optimum growth of microalgae [151]. When microalgae and bacteria require organic carbon under mixotrophic or heterotrophic circumstances, carbon might be a limiting factor [56]. Most domestic wastewaters have a low C/N/P ratio compared to algal biomass composition, which limits nutrient removal due to carbon limitation [41,152]. To overcome the limitation, external addition of carbon by aeration or through the addition of carbon dioxide could enhance the biomass productivities and effective removal of nutrients from wastewater [153]. The N:P molar ratio of more than 30:1 leads to phosphorus limitation, and the N:P molar ratio of around 5:1 leads to the nitrogen limitation [149]. The composition of domestic wastewater has been summarized in Table 5. It can be observed that most of the domestic wastewaters are carbon deficient. The microalgae are grown in wastewater often get exposed to more than two orders of nutrients found in natural sources; hence the growth can be limited by carbon and light [149].

**Table 5.** Characteristics of domestic/municipal wastewater

Wastewater type	pH	Carbon (mg/L)	Nitrogen (mg/L)	Phosphorus (mg/L)	Reference
MWW	7.4	816 ± 129 (COD)	110 ± 16 (TN)	15.3 ± 1.3$\left({PO}_{4}^{3-}-P\right)$	[278]
MWW	7.2 ± 0.5	245.6 ± 16 (TOC)	101.3 ± 2.8 (TN)	5.2 ± 1.3$\left({PO}_{4}^{3-}-P\right)$	[126]
DWW	7.6 ± 0.3	411 ± 156 (COD)	37.0 ± 9.3 (TKN)	9.2 ± 2.6 (TP)	[279]
MWW	7.04	460 (COD)	14.6 (TKN)	6.54 (TP)	[280]
DWW	7.4 ± 0.2	93 ± 12 (COD)	34 ± 5 (TN)	23.5 ± 2.8$\left({PO}_{4}^{3-}-P\right)$	[281]
DWW	7.4 ± 0.15	430 ± 198 (COD)	60 ± 11 (TKN)	8.7 ± 1.6 (TP)	[282]
DWW	7.5 ± 0.1	390.6 ± 25 (COD)	55.5 ± 5.1 (TN)	9.5 ± 0.8 (TP)	[283]
DWW	7.7 ± 0.1	494 ± 21.9 (COD)	21.6 ± 2.0 (TN)	7.1 ± 0.7$\left({PO}_{4}^{3-}-P\right)$	[284]

MWW – Municipal wastewater; DWW – Domestic wastewater; COD-Chemical oxygen demand; TOC-Total organic carbon; TN-Total Nitrogen; ${PO}_{4}^{3-}-P$ – Phosphate; TP-Total Phosphorus; ${NH}_{4}^{+}-N$-Ammonia Nitrogen; TKN-Total Kjeldahl Nitrogen

### 4.4 External aeration

Several studies have shown that the photo-oxygen generated by algal biomass is sufficient enough for heterotrophic bacterial oxidation of organic matter under no-aeration conditions, and satisfactory carbon removal has been achieved [154,155]. However, contradicting this observation, other researchers have shown that the addition of external aeration improves the removal efficiency [54]. Kang et al. [146] have reported in their study that under the limiting light condition, additional external aeration enhanced the BOD removal from 64.4 to 98.9% and the ammonical nitrogen removal from 64.4% to 98.9 %. Usually, domestic wastewater contains a low C/N ratio compared to that of algal biomass; therefore, nutrient removal can be enhanced by the addition of external aeration, which supplements the carbon required in the form of carbon dioxide [156]. However, whether the composition of wastewater necessitates the requirement of aeration or not, it is required to control the pH of the system. Else, suitable pH neutralization mechanisms need to be employed. Microalgae consume carbon dioxide, which tends to increase the pH of the system by more than 10. High pH values adversely affect the performance of algal-bacterial systems [21]. The CO_2_ addition would prevent the mixed liquor pH rise due to photosynthetic activity and thereby reducing ammonia nitrogen losses through stripping and phosphorus precipitation. Hende et al. [157] found that increasing the inorganic carbon/organic carbon ratio would improve the algal growth rate. The aeration helps to keep the algae-bacterial biomass in suspension mode. The turbulence caused by aeration helps in better substrate exchange and in the formation of algal-bacterial granules which have high settling characteristics [54].

## 5. Micropollutant or ‘emerging contaminant’ removal in algal-bacterial systems

The occurrence of micropollutants or ‘Emerging Contaminants’ (EC) in water bodies is a matter of increasing environmental concern around the world. Micropollutants present in an aquatic environment in ng/L – µg/L levels and consist of a wide range of organic and inorganic compounds which have anthropogenic as well as natural sources. Sources of man-made micro-pollutants are diverse. Inadequately treated industrial effluents, surface run-off, illegal dumping (more relevant to developing nations) are the main sources of micropollutants in the aquatic environment. Domestic wastewaters also contribute significantly to micropollutant contamination since most of the antibiotics and pharmaceutical products eventually leave the human body through excretion [158].

Micropollutants have a harmful, carcinogenic, and mutagenic effect on the life forms exposed to them over time. The anthropogenic micropollutants include pharmaceutical and personal care products, industrial chemicals, steroids & hormones, bulk pharmaceuticals, insecticides, pesticides, herbicides, heavy metals, endocrine disruptors, surfactants, and disinfection by-products [158].

**Figure 4.**
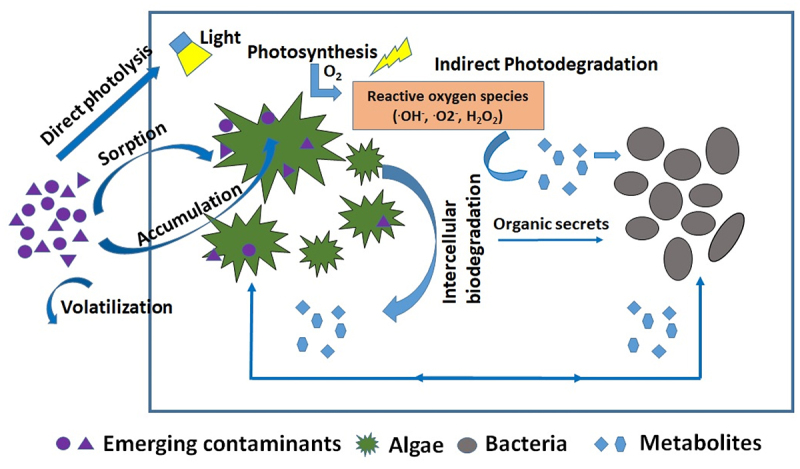
Schematic representation of micropollutants degradation pathway in algal-bacterial systems.

The conventional wastewater treatment systems are not specifically designed for micropollutant removal. As a matter of fact, the guidelines for designing wastewater treatment plants (WWTP) do not discuss anything on micropollutants. Several advanced treatment techniques for EC removal have been tried, which include advanced oxidation processes, chemical precipitation, etc. But these methods have limitations as these are energy-intensive, require separate treatment units, and the sludge disposal can also be problematic. Algal-bacterial systems have been found to be an attractive alternative. Several studies have shown that the microalgae are very efficient in removing the EC of concern [4,159–162]. Microalgae remove EC by sorption, biodegradation, photodegradation, and volatilization [4,163]. The schematic illustration of different mechanisms involved in EC removal by algal-bacterial systems has been shown in Figure 4.

### 5.1 Sorption

Sorption is the process of transferring a compound from an aqueous phase to a solid phase via surface adsorption or absorption via accumulation. Adsorption is the process of adhesion of soluble substances onto suitable physical surfaces. The sorption of EC onto any solid surface depends on the properties of the EC and solid surfaces, such as hydrophobicity, hydrophilicity, availability of functional groups, and their structure. In microalgae, the cell wall consists of polymer substances that are similar to cellulose and hemicellulose [5]. The presence of carbonyl, phosphoryl, and amine functional groups on the cell wall makes the algal cell negatively charged [5]. This causes cationic surface pollutants to be attracted towards the surface of the algae by electrostatic interaction. In addition to the properties of EC, the adsorption of EC on microalgal cells is highly dependent on the prevailing environmental conditions such as pH, temperature, and ionic strength of the solution [163]. For example, Matamoros et al. [4] have reported that the micropollutant removal efficiency was higher during the summer than during the cold season. Pharmaceutical compounds having high hydrophobicity (log K_ow_ > 4) were reported to be showing high sorption affinity on algal biomass [4]. In a recent study, Prosenc et al. [164] reported that the bisphenol compounds with higher K_ow_ were removed abiotically by adsorption, whereas bisphenols with a lower log K_ow_ were eliminated mostly through biodegradation.

Unlike extracellular adsorption, accumulation is an intracellular process that removes the pollutant from the aqueous phase by assimilating it into the cell structure. Accumulation acts as the preliminary step for enzymatic biodegradation. Few of the ECs are highly resistant to photolysis and biodegradation. However, they can be removed by algal uptake. Bai and Acharya [165] have reported that the pharmaceuticals carbamazepine and triclosan were removed mainly by algal uptake only. Godos et al. [166] also showed that biosorption was the dominant pathway in tetracycline removal during the batch assay studies having the algal biomass from HRAP in the absence of light. Bai and Acharya [167] have reported that the triclosan removal in microalgal culture *Nannohloris sp*. was mainly due to bioadsorption and bioaccumulation.

### 5.2 Volatilization

Volatilization is the process of transfer of compounds of interest from the aqueous phase to the gaseous phase. The compounds which are nonpolar in nature and having weak intermolecular forces are prone to volatilization. The compounds having a high Henry<apos;>s Law constant are prone to volatilization, and the overall loss depends on the intensity of mixing and aeration provided [168]. The volatilization potential in algae-based treatment systems is well proven for fragrances such as galaxolide and plasticizers such as octylphenol, nonylphenol, bisphenol-A (BPA), and tributyl phosphate [4,169]. Longer residence times and larger air-water interfaces available due to photooxygen generated during photosynthesis facilitate the volatilization.

### 5.3 Photodegradation

Algae-based processing technology uses light energy to perform photosynthesis, facilitating a unique micropollutant removal mechanism named photodegradation. Photodegradation is a well-reported micropollutant removal mechanism in natural environments exposed to sunlight [170]. Few EC compounds such as ciprofloxacin and triclosan are photosensitive [165]. Based on the compounds involved, the process of photodegradation can be either direct or indirect photodegradation [171]. In direct photo-transformation, the change in chemical structure is caused by the impact of a photon that directly hits the contaminant and induces bond breaking, photoionization, or transformation into reactive excited states [172]. The pollutants having aromatic rings, heteroatoms, conjugated Π systems, and other functional groups are susceptible to direct photolysis [173]. Fluorescent compounds such as antibiotics degrade through direct photolysis, during which the photons from the light induce covalent modification and chemical damage, a process known as photo-bleaching [163]. Antibiotic compounds such as tetracycline, cefazolin, ciprofloxacin, and cephapirin are highly susceptible to photolysis in microalgal based wastewater treatment systems [174]. Norvill et al. [175] have found that 40% more tetracycline can be removed during the daytime compared to the nighttime due to the availability of natural sunlight. The degree of photo-transformation is dependent on variables such as light intensity, duration, wavelength spectrum, pH, temperature, dissolved ions matrix, and suspended solids concentration [163]. The high concentration of suspended solids in the algal reactor can adversely inhibit the photo-transformation process [166]. The dissociation constants (pKa) and pH of the medium decide whether a particular compound is in an ionic state or a molecular state. For example, triclosan in anionic form is a hundred times more degradable than its molecular form [176].

The EC removal can be enhanced through indirect photo-transformation when the microalgae are present in the system. During indirect photo-transformation, photon strikes a secondary compound called a photosensitizer, making it a reactive transient species, often as a radical. These radical species interact with the compounds of interest and degrade them. Extremely diverse photosensitive compounds are present in wastewater, such as nitrates, nitrites, humic substances, iron, and dissolved oxygen [163]. Reactive oxygen species (ROS) are generated during the respiration and photosynthesis process in algae and plants (Figure 4). Microalgae produce excess ROS species such as superoxide radical (O_2_^−^), singlet oxygen (O_2_), and hydrogen peroxide (H_2_O_2_) under environmental stress conditions [177]. Bai and Acharya [167] have shown that nitrate and humic acids present in the wastewater promoted the tetracycline degradation in the algal reactor by producing free radicals. The organic compounds excreted by microalgae, such as biopolymers of polysaccharides and proteins, could act as photosensitizers in the microalgae-based systems, promoting the photodegradation of micropollutants [178]. In algae-bacterial treatment systems, light illumination is a must for algal growth. Therefore, photodegradation can also play a vital role in EC removal in algal-bacterial systems.

### 5.4 Biodegradation

Biodegradation of the contaminants happens during the catabolism process in which the microbes break the organic compounds by releasing energy. Microalgae are highly adaptive microorganisms that can survive in harsh environments by switching between autotrophic, heterotrophic, and mixotrophic metabolism [179]. It was reported that microalgae are very efficient in EC removal. Matamoros et al. [4] evaluated the removal of ECs such as ibuprofen, caffeine, galaxolide, 4-octylphenol, tributyl phosphate, and carbamazepine fed to the algal batch reactors having *Chlorella* and *Scenedesmus* sp. as the dominant species. It was shown that biodegradation was the major removal mechanism for caffeine, ibuprofen, and carbamazepine removal. They have reported that microalgae enhanced the ibuprofen removal by 40%, and the lag phase of caffeine removal was reduced by three days. The EC removal depends on the algal species involved. *Chlorella* species was reported to be very efficient in the removal of several classes of antibiotics [174,180]. *Chlorella sorokiniana* was able remove 60–100% of ibuprofen, diclofenac, metoprolol and paracetamol [181]. In a very recent study, Peng et al. [182] evaluated the removal of antibiotic levofloxacin using microalgal species *Chromochloris zofingiensis* in phototrophic and heterotrophic conditions. The maximum removal efficiency of levofloxacin was obtained in heterotrophic conditions. The removal was attributed to bioaccumulation (8%) and biodegradation (>90%). The possible reaction mechanisms involved hydroxylation, defluorination, oxidation, ring cleavage, demethylation, decarboxylation, denitrification, and dehydrogenation.

In algal-bacterial systems, the interaction between algae and bacteria is not restricted to the exchange of carbon dioxide and oxygen but also includes the complementary exchange of metabolites [183]. The oxygen produced by the microalgae during photosynthesis helps in the growth and respiration of bacteria to degrade the pollutants present in the wastewater. As illustrated in Figure 4, the EC biodegradation starts with the sorption of pollutants onto the microalgal cell surface. The pollutants get absorbed and accumulated into the algal cells. The accumulated pollutants get biotransformed or bioaccumulated through intercellular enzymatic reactions by releasing some organic secretes. These organic secrets or metabolites produced by microalgae can be uptaken by bacteria. The microalgae are highly tolerant to antibiotics than the heterotrophic bacteria as antibiotics are specifically designed to kill the bacteria. Hence, the algal-bacterial symbiotic relationship can enhance antibiotic removal. For instance, Ismail et al. [184] have shown that *Chlorella* sp. mixed with bacterial consortium enhanced ketoprofen removal. Matamoros et al. [185] also reported that more than 99% caffeine removal was observed in combined algal-bacterial systems than only 17% removal in sole microalgal incubation. The organic exudates released by microalgae aided in the caffeine biodegradation by bacteria. Prosenc et al. [164] studied the removal of 28 ECs (pharmaceuticals, bisphenols, neonicotinoids, and selected transformation products) in monoculture of *Chlorella Vulgaris* and mixed algal-bacterial culture. They reported that the mixed algal-bacterial culture performed better at removing bisphenols than algal monoculture. The bisphenols having low log K_ow_ were mainly removed through biodegradation. In a recent study, Li et al. [186] showed that algal-bacterial symbiosis played a major role in treating anthraquinone dye wastewater. They have investigated the anthraquinone removal mechanism by comparing the algae treatment followed by bacterial treatment and bacterial treatment followed by algal treatment. It was observed that the algal treatment followed by bacterial treatment showed high anthraquinone removal. This was attributed to the fact that algal species *Chlorella* was able to break the molecular bonds of anthraquinone and convert it into intermediate molecules. These intermediate molecules were completely uptaken by the heterotrophic bacteria. The symbiotic relationship of algae and bacteria considerably enhanced the anthraquinone removal.

In algal-bacterial systems, the interaction between different species and co-metabolism may increase the transformation rate of the contaminant. Microalgae release organic compounds such as carbohydrates and amino acids which can act as substrates for co-metabolism of organic compounds during bacterial heterotrophic metabolism. In turn, the bacteria facilitate the nutrients regeneration, vitamins, and phytohormones promoting algal growth [168]. For example, the addition of microalgae to bacterial inoculum improved the transformation rate and reduced the lag phase for ibuprofen and caffeine removal [185]. Studies have demonstrated that the degradation of the contaminants can be promoted by the addition of organic substances or nutrients [187–189]. For instance, Xiong et al. [190] demonstrated that adding sodium acetate as an electron donor significantly increased the ciprofloxacin removal efficiency from 13% to 56% because of co-metabolism.

Surfactants hold a significant proportion of domestic wastewater contaminants. Linear Alkaline Sulfonate (LAS) anionic surfactant is a significant component in detergents and cleansing agents. In a recent study, it was shown that LAS and caffeine removal was more algal-bacterial trickling photobioreactor in comparison to bacterial trickling filter treating the same wastewater [126]. In this study, the possible mechanism of removal was observed to be photodegradation and biodegradation for LAS and caffeine, respectively. The schematic illustration of the mechanism for photosynthetic enhanced degradation is shown in Figure 4. Various studies on micropollutants removal in algal-bacterial systems have been summarized in Table 6. In a recent study, Avila et al. [191] have evaluated the pesticides acetamiprid, propanil, and bentazone removal in a pilot-scale tubular photobioreactor. They have reported that acetamiprid and propanil were effectively removed by microalgae, and the removal was mainly due to the algal-mediated biodegradation. However, bentazone was not removed as it was recalcitrant. The maximum removal efficiencies were obtained during steady-state for propanil (99%) and acetamiprid (71%). Enhanced micropollutants removal can be achieved in algal-bacterial systems due to photodegradation and symbiotic interactions between algae and bacteria.

**Table 6.** Micropollutant removal efficiencies and mechanism of removal in algal-bacterial system

Algal species	Treatment system and wastewater type	Micropollutant & Removal efficiency %	Mechanism of removal	Reference
*Scenedesmus obliquus* *Chlorella vulgaris*	^a^ Lagoon water	Ibuprofen – (60) Triclosan – (100)	Biodegradation Phototransformation	[285]
*Chlamydomonas mexicana*	^a^ BBM media	Ciprofloxacin-(56)	Biodegradation	[190]
Mixed algal-bacterial culture	Trickling Filters SWW	LAS – (99–95.6) Caffeine – (96.3–86.2)	Phototransformation Biodegradation	[126]
Mixed algal-bacterial culture	Anoxic-aerobic photobioreactor DWW	Ibuprofen – (94 ± 1) Triclosan – (100 ± 0) Naproxen – (52 ± 43) Salicyclic acid – (98 ± 2) Propylparaben – (100 ± 0)	Biodegradation Bioaccumulation Sorption	[159]
*Nannochloris sp*.	^a^ Lake water	Sulfamethoxazole – (40) Ciprofloxacin – (100) Triclosan – (100)	Photolysis Biodegradation	[165]
*Chlorella pyrenoidosa*	Algae activated sludge combined system BG 11 media	Cefradine – (89.9) Cephalexin – (94.9) Ceftazidime – (89.7) Cefixime – (89.7)	Photodegradation Biodegradation	[177]
*Chlorella sp*. *Scenedesmus sp*.	Aerated batch reactors Urban wastewater	Caffeine – (99) Ibuprofen – (60) Galaxolide – (99) Tributyl phosphate – (99) 4-octylphenol – (99) Tris(2-chloroethyl) phosphate < (20) Carbamazepine < (20)	Volatilization Biodegradation	[4]
*Chlorella sp*. *Nitzschia acicularis*	2.5 L reactor Secondary wastewater	Bisphenol A – (46) Bisphenol AF – (80) Bisphenol F – (87) 2,4-dichlorophenol – (76)	Biodegradation Bioadsorption	[286]
*Chlorella vulgaris* *Scenedesmus sp*., *Westella botryoides*	HRAP Sewage wastewater	Hormones – (7–55) Pharmaceuticals – (17–54) Xenoestrogens – (41–53)	Bioadsorption Biodegradation Photodegradation Volatilization	[287]
Mixed algal culture	HRAP Urban wastewater	Acetaminophen – (99) Naproxen – (89) Caffeine – (98) Carbamazepine – (62) Ibuprofen – (99) Galaxolide – (97) Methylparaben – (75) Triclosan – (95) Celestolide – (53) Atrazine – (85) Diclofenac – (92) Biophenol A – (85)	Bioadsorption Biodegradation Photodegradation Volatilization	[185]
Mixed algal culture Diatom+Bacteria Algae+Bacteria	^a^ Laundry wastewater	Caffeine – (89.7) Cumene hydroperoxide – (84.7) LAS – (61.6) Disulfoton-sulfone – (53.9) Hexazinone – (82.3) 4-Nitrophenol – (96.6) Caffeine – (87.3) Cumene hydroperoxide – (81.3) LAS – (100) Disulfoton-sulfone – (100) Hexazinone – (100) 4-Nitrophenol – (100) Caffeine – (87.9) Cumene hydroperoxide – (52.1) LAS – (100) Disulfoton-sulfone – (100) Hexazinone – (100) 4-Nitrophenol – (70.9)		[288]

Where: ‘^a^’ – Lab study

## 6. Algal-bacterial systems modeling

Modeling is the process of a representation of a process in mathematical form. The reactions that take place in algal-bacterial systems are complex compared to the conventional bacterial wastewater treatment process. Mathematical modeling offers an advantage to investigate the effect of different factors on the system process, thus, contributing to the optimization of design and operational control parameters [192]. In comparison to conventional treatment processes, very little is known about how the algae-bacterial systems work, particularly with regard to interactions between the algae and bacteria [61,72]. Several bacterial mathematical models are well established and currently applied in real-scale treatment systems [193,194]. The most widely used models for the modeling wastewater treatment systems are Activated Sludge Models ASM1, ASM2, ASM3 developed by IWA [195]. ASM3 was the latest version which describes decay processes and cell internal storage compounds [195]. The ASM3 model represents nitrification and denitrification as a single step process. The bacterial activities of ammonia-oxidizing bacteria (AOB) and nitrite-oxidizing bacteria (NOB) are not clearly distinguished. The model was extended to consider two-step nitrification and two-step denitrification with nitrite as an intermediate compound by Iacopozzi et al. [196] and Kaelin et al. [197]. Several researchers have modeled the different microalgae processes [198–200]. Modeling of the algal-bacterial systems has to consider several factors such as light, carbon limitation, ammonia stripping along with biological and hydrodynamic processes. Arashiro et al. [289] modeled nitrogen removal by modifying ASM3 by including two processes pertaining to algal growth and endogenous respiration. Ariza [201][] extended the model developed by Arashiro et al. [289] by incorporating two additional processes: nitrogen storage by algal biomass and phototrophic growth on stored nitrogen. These process parameters were estimated by performing respirometer tests on the photo-activated sludge. Different algal-bacterial models available, their features, processes, components, and their limitations have been summarized in Table 7.

**Table 7.** Features, processes and components adapted in algal-bacterial system models

Model	Basic Model	Simulation platform	Processes	Components	Limitations	Experimental validation
Solimeno et al. [199]	RWQM1	COMSOL Multiphysics	10 processes Microalgal processes: Growth on ammonia, nitrate, endogenous respiration, inactivation, chemical equilibrium and gas transfer	10 components microalgae biomass (X_ALG_), ammonium nitrogen (S_NH4-N_), ammonia nitrogen (S_NH3_), nitrate(S_NO3-N_), dissolved oxygen(S_O2_), bicarbonate(S_HCO3_-), carbon dioxide (S_CO2_),, carbonate(S_CO3_), hydrogen ion (S_H_), hydroxide ion(S_OH_)	Light attenuation, bacterial processes are not included. Hydrodynamic flow and transport equations need to be coupled	Model was calibrated with a batch mesocosm algal culture having surface area 1.30 m^2^ and 0.55 m depth
Zambrano et al. [202]	Bacterial dynamics – ASM1 Algal dynamics – Solimeno et al. [199]	MATLAB/ Simulink	6 Processes Algal growth on NH4, NO3; algal decay; bacterial growth and decay; oxygen transfer	8 components Biomass population: algae (X_alg_), bacteria (X_bac_), dissolved substrates: nitrate(S_NO3_), ammonium (S_NH4_),, dissolved gases: carbon dioxide (S_co2_), oxygen (S_o2_).	pH dynamics not considered; algal inhibition by excess light and excess carbon dioxide is not included	Calibrated with 2 lab scale 1 L PBRs treating sewage in batch mode.
Arashiro et al. [289]	Bacterial dynamics – Modified ASM3[196] Algal dynamics – their own input	Aquasim 2.0	23 processes Algal processes: Growth and endogenous respiration Bacterial processes: aerobic growth, endogenous respiration, anoxic growth and endogenous respiration	17 components Soluble inert organics SI, ammonium (S_NH4_), nitrate(S_NO3_), nitrite(S_NO2_), nitrogen gas(N_2_), dissolved oxygen(S_O2_), readily degradable substrate(S_S_), AOB biomass(X_AOB_), NOB biomass(X_NOB_), inert particulate organics(X_I_), heterotrophic biomass(X_H_), phototrophic biomass(X_P_), organics stored by heterotrophs(X_STO_)	Very limited algal processes considered	Calibrated and validated the model with experimental data from 2 L PSBR operated at 4d HRT treating centrate wastewater
BIO-ALGAE model [208]	Bacterial dynamics – Modified ASM3[196] and Algal dynamics – [208]	COMSOL Multiphysics	25 processes Algal processes: Growth on S_NH4,_ S_NO3,_ endogenous respiration and decay. Bacterial processes: Aerobic growth on S_NH4,_ S_NO3,_ anoxic growth on S_NO3,_ S_NO2,_ endogenous respiration, decay Nitrifying activity: Growth, endogenous respiration and decay of X_AOB and_ X_NOB,_ Hydrolysis and chemical equilibrium	19 components Algal biomass (X_ALG_), heterotrophic bacteria (X_H_), nitrite oxidizing bacteria(X_NOB_), ammonia oxidizing bacteria(X_AOB_), slow degradable organic matter(X_S_), inert particulate organic matter(X_I_), ammonium nitrogen (S_NH4-N_), ammonia nitrogen (S_NH3_), nitrate(S_NO3-N_), nitrite(S_NO2-N_), phosphate(S_PO4-_), dissolved oxygen(S_O2_), carbon dioxide (S_CO2_), bicarbonate(S_HCO3_-), carbonate(S_CO3_), hydrogen ion (S_H_), hydroxide ion(S_OH_), Inert soluble organic matter (S_I_), readily biodegradable soluble organic matter (S_S_)	CFD coupling can help to predict more accurate pH dynamics, dissolved oxygen profiles and other components profiles	Model was calibrated and validated with the data from two HRAPs (3.5 m^2^, 0.3 m depth) operated at 4.5d HRT treating municipal wastewater
ASM-A [203]	Algal dynamics – Modified ASM-2d with their own inputs	MATLAB	6 processes Algal uptake and storage of nitrogen and phosphorus; photoautotrophic growth; heterotrophic growth; decay	11 components Soluble ammonium nitrogen (S_NH4_), Soluble nitrate+ nitrite nitrogen (S_NO_), Inorganic soluble P (S_PO4_), Internal cell quota N (X_AlgN_), Internal cell quota P (X_AlgPP_), Alkalinity S_Alk_, Dissolved oxygen S_O2_, Carbon source S_A,_ Slowly biodegradable organic matter X_S_, Algal biomass concentration X_Alg_, Non-biodegradable organic matter X_I_	Bacterial processes are neglected; Factors such as light attenuation, toxicity, photo-oxidative damage, temperature effects are not considered	Model was calibrated and validated with data from 24 L airlift PBR operated in SBR mode with synthetic medium
Casagli et al. [212]	MRWQM1 [290] BioAlgae2 [291]	Aquasim 2.0	19 processes Algae processes: Growth on S_NH_, S_NO3_ Heterotrophic bacterial processes: Aerobic – Aerobic respiration, decay, growth on S_NH_, S_NO3_, respiration Anoxic – growth on S_NO2_, S_NO3_, respiration, hydrolysis Nitrifying bacteria: Aerobic X_AOB_ – ammonification, decay, growth, respiration, X_NOB_ – growth, respiration, decay.	17 components Phototrophic algae (X_ALG_), Ammonium oxidizing bacteria (X_AOB_), Nitrite oxidizing bacteria (X_NOB_), Heterotrophic bacteria (X_H_), Slowly biodegradable organic matter (X_S_), Readily biodegradable particulate matter (S_S_), Inert particulate matter (S_I_), Total inorganic carbon (S_IC_), organic nitrogen (S_ND_), Total ammoniacal nitrogen (S_NH_), Nitric nitrogen (S_NO3_), Nitrous nitrogen (S_NO2_), Total inorganic phosphorus (S_PO4_), Nitrogen gas (S_N2_), Water (S_H2O_), Dissolved oxygen (S_O2_)	n.a.	Model was set up and calibrated with the data from a 56 m^2^ raceway pond treating synthetic wastewater

‘n.a’ – not availble

A simple model has been developed by Zambrano et al. [202] for describing the algal-bacterial interactions in a photobioreactor. The bacterial processes in the model were adapted from the Activated sludge model (ASM1), and algal process dynamics were adapted from the algal mechanistic model presented by Solimeno et al. [199]. This developed model was implemented in MATLAB Simulink platform by considering 6 processes and 6 components, as shown in Table 7. The sensitivity analysis of the model indicated that the maximum algae and bacteria growth rate half-saturation constant for carbon and bacteria growth yield was being the most sensitive parameters. The model was able to predict the concentration of ammonia and nitrate accurately. However, the overestimation of dissolved oxygen beyond the saturation concentration was attributed to not considering the effect of light attenuation and pH dynamics in the model. In another study, Wágner et al. [203] developed the ASM-A model as an addition to the ASM-2d model for simulating algal growth in HRAP and PBRs. The model was executed in MATLAB by considering 6 processes and 11 components, as shown in Table 7. The model was validated using the independent data obtained from 24 L photobioreactor. The nutrient limitations are considered according to Droop formulation, inorganic carbon uptake by Monod kinetics, and the light limitation was incorporated as per the model developed by Béchet et al. [204].

Solimeno et al. [205] developed and implemented the BIO_ALGAE model in the COMSOL-Multiphysics platform. The model was developed by coupling RWQM1 [206], ASM3 [196], and Solimeno et al. [199]. This model is applicable to PBRs and HRAPs. The model considered 19 variables with 25 physical, chemical, and biological processes, as mentioned in Table 7. The C, N, P limitation was included by using Monod-type kinetics. The main characteristic of this model was the incorporation of carbon limiting growth for microalgae and nitrifying bacteria. The temperature dependence of the microalgae was included by Arrhenius equation, and the light intensity effect on algal photosynthesis was included from the dynamic model reported by [207]. The calibration and validation of the model were performed using the experimental data from two HRAPs treating real wastewater [208]. Some of the new features incorporated by the model were carbon limitation on microalgae growth, photorespiration, light attenuation, temperature, and pH dynamics. The sensitivity analysis of the model shows that light is the most sensitive parameter for algal growth. Light intensity is regarded to be the most important limiting factor in microalgal systems [209]. The results indicate that the model was able to predict the nutrient levels, pH variation, dissolved oxygen, and biomass concentrations accurately. Shriwastav et al. [210] developed a comprehensive mechanistic model for simulating algal-bacterial dynamics in a photobioreactor. The developed model includes 37 state variables covering all the physico-chemical and biological processes in the system. In a recent study, Yang et al. [211] developed an algal-bacterial system model by extending the ASM3 model with modified algal biokinetics. The model predicted that a satisfactory level of treatment efficiency could be achieved with an algal-bacterial biomass concentration of 1 g/L within eight hours under non-aeration conditions.

Most of the existing algal-bacterial models only evaluated the systems for a shorter duration. Recently, Casagli et al. [212] have developed a comprehensive model ALBA for describing the long-term dynamics of the algal-bacterial ecosystem in a raceway pond of 56 m^2^ treating wastewater. The model has considered 19 processes and 17 components based on biological, physical, and chemical parameters, as shown in Table 7. Compared to the other models, the ALBA model was used to evaluate the algal-bacterial systems for a longer duration by considering the data of 443 days of operation. The model was found to be capable of simulating both the long-term seasonal dynamics and short-term nycthermal dynamics. The Monod kinetics adopted by the ASM models for nutrient limitations are reported to be overestimating growth limitation when multiple limiting nutrients are available. The ALBA model used minimum law to incorporate multi-nutrient limitations. They have reported that the developed model can support the implementation of smart control techniques such as paddle wheel velocity control by balancing the aeration, mixing, and degassing effects. In another study, Sheng et al. [213] developed an algal-bacterial model based on ASM3 model by incorporating processes related to heterotrophic bacteria and algae. The model was able to predict the profiles of dissolved oxygen and pollutant removal in a photo-sequencing batch reactor accurately. Manhaeghe et al. [214] have analyzed the carbon fluxes in algal-bacterial flocs under different growth conditions (photoautotrophic, mixotrophic, and heterotrophic) using a model developed based on respirometric-titrimetric data. The model was able to take into account the EPS production and consumption and accurately predict the heterotrophic bacterial growth and algal growth under photoautotrophic, mixotrophic, and heterotrophic conditions.

Most of the algal-bacterial models assumed ideal mixing conditions in reactors which is not true in real scale systems. Computational Fluid Dynamics (CFD) simulation will provide a detailed insight understanding of the clear hydrodynamic pattern of the system. The integration of the developed models with the CFD platform can help to predict the accurate biochemical process parameters like pH, dissolved oxygen, and other components [192]. In full-scale systems, the components are not distributed uniformly, leading to optical absorption and light shading of the cells. Coupling CFD simulation with the distribution of local light intensity could reproduce the light intensity history of microalgae, called as flashing effect, which has a considerable effect on photosynthetic activity [215].

## 7. Biomass harvesting

The major challenge for the microalgal application in wastewater treatment is the separation of algae from effluent after the treatment process [50]. Microalgae have a small size (2–20 μm in diameter), relatively low biomass concentration (around 1–5 g/L) because of limitation of light penetration, and have a similar density as water. All these factors make algae harvesting troublesome at industrial scale applications [216]. Algae harvesting alone contributes to around 20–30% of production costs depending on the species diversity, culture conditions, and cell density [217,218]. The algal biomass grown in wastewater has to be separated before discharging the treated water. The harvested biomass can be used for resource recovery. The harvesting methods can be mechanical, chemical, electricity-based, or through biological means [36]. Gutiérrez et al. [219] evaluated the coagulation-flocculation with natural flocculants, Ecotan and Tanfloc, for harvesting microalgae from an HRAP treating urban wastewater. The results have shown that more than 90% recovery was achieved within 10–20 min duration. In another set of studies, Markeb et al. [220] used magnetite-based nanoparticles as adsorbents for harvesting microalgae *Scenedesmus sp*. treating real wastewater. More than 95% harvesting efficiency was achieved within 27 minutes of the contact time. The sedimentation process is the most primary and inexpensive way of harvesting process. In a recent study, Leite and Daniel [221] explored the way of optimizing the sedimentation parameters for *Chlorella Sorokiniana* harvesting by inducing high pH. More than 98% efficiency was achieved when the velocity gradient was 250 s^−1^, 10 sec mixing time, and the pH was 12. Even though higher biomass harvesting efficiencies can be achieved, these mechanical, electrical based separation techniques are energy-intensive. Chemical based techniques leave behind certain chemical byproducts, which hinder further biomass processing for resource recovery.

Microalgae harvesting by using bio-flocculation has several advantages, such as zero toxicity, higher efficiencies, and less energy consumption. Bio-flocculation can happen due to the interactions between algae-bacteria, algae-fungus, and algae-algae [222]. The bioflocculation process is induced by the extracellular polymeric substances secreted by the species involved. In algae-bacterial systems, bacterial communities play a pivotal role in the formation of aggregates. Based on the operating conditions, the resulting algae-bacterial granules will have a size of 100–5000 μm range, a hundred times bigger than the microalgae cells [131]. Lee and co-workers [223] demonstrated the key role played by the microalgal-associated bacteria in *Chlorella Vulgaris* flocculation. In their study, it was stated that the axenic culture of *Chlorella vulgaris* has only 2% flocculation activity compared to algal culture having associated with bacteria which has a flocculation efficiency of around 94%. In another study, Nguyen et al. [224] showed that *Chlorella Vulgaris* cultured with bacteria associated with wastewater attained a flocculation activity of > 92% by bio-flocculation. Light intensity is also a crucial factor in the production of extracellular polymeric substances, which helps in the granules formation. The high settling velocity of algal-bacterial aggregates makes biomass harvesting simple in algal-bacterial systems.

## 8. Resource recovery

**Figure 5.**
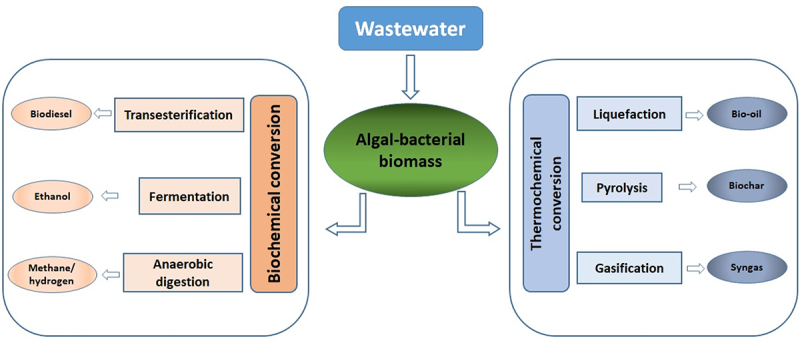
Conversion technologies for biofuel production from algal-bacterial biomass [225].

Numerous studies have reported that a variety of value-added products and biofuels can be extracted from the algae-bacterial biomass grown during wastewater treatment. Also, clean biofuels – methane and hydrogen can be generated from algae-bacterial biomass through anaerobic digestion. Different aspects of biofuels production from the algal-bacterial biomass have been shown in Figure 5. The conversion of algal-bacterial biomass into a valuable resource depends on biomass type, technology adapted, and end-use. The algal-bacterial biomass can be converted to biofuel by either biochemical or thermochemical conversion [225].

### 8.1 Biochemical conversion

Biochemical conversion of biomass includes transesterification, fermentation, and anaerobic digestion. Biodiesel can be produced from the lipid-containing biomass via transesterification. The microalgal biomass grown on wastewater have a lipid content between 10 and 30% of the dry weight [110]. Recently, Katam and Bhattacharyya [226] have used immobilized and suspended microalgae and bacterial sludge cultures for bio-lipid synthesis. Higher lipid content of 40% was reported for the immobilized cultures, whereas only 16% lipid content was reported for the suspended cultures. The lipid accumulation in the microalgal species depends on several associated factors such as species involved, nutrient availability, and operating conditions. In a separate study, Katam and Bhattacharyya [227] showed that solids retention time (SRT) has an effect on the lipid content of the biomass. They have reported that the lipid content was decreased with an increase in solids retention time. This was attributed to the nutrient-deprived stress conditions at low solid retention times. Lipid productivity of more than 20 times can be obtained in heterotrophic metabolism compared to photoautotrophic metabolism. In algal-bacterial consortia, the bacterial association enhances the lipid accumulation in microalgae. For example, adding bacterial species *Azotobacter Chroococcum* to the microalgae, *Chlamydomonas reinhardtii* increased the lipid production by 2.3 times compared to nutrient depletion conditions [228]. This increase in lipid production might be attributed to the change in the activity of the lipid accumulating enzymes. The nutrient limitation also triggers lipid accumulation in the microalgal cells. Under nutrient deprived conditions, a major part of the available carbon is converted to lipid instead of carbohydrates and proteins [229]. Table 8 summarizes the studies on wastewater treatment using microalgae, highlighting the microalgal species used and their lipid content.

**Table 8.** Lipid content of various microalgae species grown on wastewater

Wastewater type	Algal species	Lipid content (% dry weight)	Reference
Anaerobic digester effluent	Mixed culture	15–40	[156]
WWTP effluent	*C. vulgaris* *Chlorella pyrenoidosa* *S. obliquus* *Scenedesmus dimorphu*	29.1 24.4 17.0 30.8	[292]
MWW	*S. obliquus*	15.9	[293]
MWW	*S.obliquus* *Chlorella sorokiniana*	23.26 ± 3.95 22.74 ± 3.11	[294]
Synthetic wastewater	*S. Obliquus*	11–14	[295]
MWW	*Scenedesmus sp., Chlorella sp*., and *Acutodesmus sp.*	12–25	[296]
MWW(secondary treated)	*Chlorella pyrenoidosa*	39	[297]
MWW	*Nannochloropsis sp., Chlorella sp., Chlamydomonas* *Reinhardtii, Scenedesmus bijugatus*, *and Oscillatoria*.	18–25	[298]
MWW	*Micractinium pusillum* *Mucidosphaerium* *Pulchellum* *Coelasteum sp*. *Pediastrum boryanum* *Desmodesmus sp*.	48.2 ± 1.8 46.3 ± 3.6 30.4 ± 0.9 40.2 ± 0.9 31.5 ± 5.8	[299]
DWW	Mixed microalgae	15–40	[226]
MWW	*Chlorella sorokiniana*	12	[300]
MWW + sea water	*Phaeodactylum tricornutum*	18.3–35.5	[301]

MWW – Municipal wastewater; DWW – Domestic wastewater

Extracting more than one type of biofuel or additional co-product from algal biomass enhances the biomass value by offsetting the environmental impacts associated with the algal biorefinery [230]. After the extraction of lipids, the residual biomass can be subjected to alcoholic fermentation for ethanol production or biogas generation via anaerobic digestion. The anaerobic digestion of the biomass and converting it into combustible CH_4_ gas is one of the promising approaches for resource recovery [104]. The operating conditions of the algal-bacterial systems, treating wastewater, affect the biochemical methanogenic potential (BMP) of the biomass. Arcila and Buitrón [231] showed a 20% increase in BMP with an increase in the SRT from 6 days to 10 days for the algal-bacterial biomass from an HRAP operating at HRT equal to SRT. The factors that negatively affect the BMP values are the presence of low biodegradable species like *Klebsormidium sp., Navicula sp., and Nitzschia sp*., and the low C/N ratio in the biomass due to the increase of protein content [131]. With the increase in the lipid content, there was an increase in the BMP [232]. After the lipid extraction from algal-bacterial biomass, the leftover biomass residue is rich in carbohydrates and proteins, which makes anaerobic digestion a possible way for resource recovery []. The ultimate methane yield from anaerobic digestion of lipid-extracted biomass residue was 296 ± 2 mL/g VS, and hydrothermal pretreatment enhanced the methane production rate by 15–30% [233].

Biohydrogen production from algal-bacterial biomass is also a potentially sustainable and energy recovery process [234,235]. Microalgae can produce biohydrogen either through fermentation or photolysis. The high contents of carbohydrates, lipids, and proteins in algal biomass make it a potential feedstock for biohydrogen production. The biohydrogen production during the fermentation depends on the available fermentative organisms and biomass pretreatment. For instance, Chen et al. [236] have recently studied the effect of gamma radiation pretreatment on biohydrogen production from microalgae *Laminaria Japonica* biomass using dark fermentation. They observed an increase in biohydrogen by 71.4 % for the gamma pretreated biomass. Batista et al. [237] have shown the feasibility of coupling the urban wastewater treatment with biohydrogen production in an integrated approach. They reported a biohydrogen production yield of 56.8 mL/g volatile solids from the dark fermentation of *Scenedesmus obliquus* grown on wastewater. Co-fermentation of algal biomass added with co-substrate in the presence of a catalyst can further enhance the biohydrogen production. For example, Srivastava et al. [238] have reported a 37.14% increase in cumulative biohydrogen production during dark fermentation of *Lyngbya limnetica* biomass added with glucose as co-substrate in the presence of Fe_3_O_4_ nanoparticles as catalyst. Even though biohydrogen production from microalgal biomass is ecofriendly, the quantity of biohydrogen produced is low for commercialization. Further research is needed on different pretreatment methods to enhance biohydrogen production.

### 8.2 Thermochemical conversion

Despite having prospects, the scaling up of algal biofuel production has been hindered by associated technical, economic, and environmental challenges [239]. Algal biofuel technologies depend on algal species and oil extraction procedures. The energy consumed by the lipid extraction process accounts for 70–90% of overall energy usage [240]. Thermochemical conversion can be a viable option for converting raw or leftover biomass into a useful product. Hydrothermal liquefaction (HTL) can convert biomass into valuable products such as biocrude. In this process, the wet algal-bacterial biomass is directly converted into liquid fuel. HTL is the process of depolymerization of biomass into biocrude under controlled temperature and pressure. HTL has been performed on the algal-bacterial biomass (harvested from wastewater bioreactors) at 300°C temperature, (10–12) MPa pressure, and 30 min reaction time [239]. The biocrude oil yield ranged from 37.9% to 52.2%. In another similar study, microalgae biomass grown on domestic wastewater, subjected to HTL at 27 bar pressure and 230°C temperature for 20 min yielded a biocrude of 43% on a dry mass basis. The biocrude was rich in ketones, aldehydes, and fatty acids [241].

Pyrolysis of the algal-bacterial biomass at a 300–700°C temperature in an inert atmosphere yields various products such as bio-oil, biochar, and syngas. Recently, converting algal biomass into biochar has gained much attention due to its application in environmental remediation [242,243]. The biochar can be used to remove the micropollutants from the aqueous phase. Recently, Nguyen et al. [244] converted the algal species *Ascophyllum nodosum* into biochar using ZnCl_2_ as a chemical activating agent. The synthesized biochar had a very high adsorption capacity (150–400 mg g^−1^) while removing ciprofloxacin. Algae is inherently rich in protein content, making the biochar rich in nitrogen without any additional chemical modification. This makes the algal-derived biochar superior to the lignocellulose-derived biochar [245].

## 9. Challenges and future prospective of the algal-bacterial system for wastewater treatment

Even though algae-bacterial systems have several advantages like nutrient recovery, reduction in aeration, and operational cost, they are yet to be used as main stream wastewater treatment systems. For algae-bacterial wastewater treatment systems to be competitive with conventional biological treatment systems following factors need to be optimized: light intensity, nutrients supply (CNP), pH, temperature, mixing conditions, algal-bacterial strains, and operational conditions such as HRT and SRT. The major limitation for algal-bacterial systems is providing sufficient light intensity for the photosynthetic process. It was reported that the light shading effect has a major effect on the biomass productivity in photobioreactors having biomass concentration more than 1 g/L [246]. Most of the algal-bacterial systems have an HRT of 2–10 days [247]. This needs to be reduced to 0.2–0.4 days to be competitive with the conventional activated sludge process. According to a recent study, by controlling the light intensity and aeration, suitable conditions for algal-bacterial growth and nutrient removal could be established [146]. In the same study, it was stated that by providing sufficient light illumination, photosynthetic oxygen production by microalgae could replace the mechanical aeration which is required to support respiration of the heterotrophic microbial consortia in the system. Most of the real-scale activated sludge systems are set up in outdoor environments. Even though they are exposed to sunlight, the indigenous microalga was not able to survive and grow due to higher biomass concentration which prevents the penetration of light. For converting existing treatment systems into algal-bacterial systems, it is required to provide the sufficient submerged light illumination for stimulating algal growth. In a very recent study, Katam and Bhattacharyya [248] have shown that nutrient removal can be enhanced significantly by introducing submersible light illumination and algal biofilms into the existing conventional activated sludge process. The algal strains that can grow under low light conditions and survive in extreme environments should be selected as algal inoculum. For example, diatom species can grow faster in low-light conditions [249] and survive under harsh environmental conditions [250].

Wastewater treatment coupling with the concept of algal biorefinery allows for more efficient utilization of the algal biomass grown on wastewater by reducing the waste component associated with a wastewater treatment system, which will help to ensure long-term economic viability. Extensive research on the downstream processing for the extraction of bioproducts/biofuel from biomass is needed to reduce the overall production cost. Research on coupling microalgae with bioelectrochemical systems (BES) has significantly increased in the past few years [251,252]. BES systems generate bioelectricity while remediating the wastewater or waste biomass. BES is a widely studied wastewater treatment technology for carbon and nutrient removal. Coupling microalgae with BES can set of the drawbacks associated with it while simultaneously enhancing the bioelectricity production. For instance, introducing the algal species, *Chlorella Vulgaris* in the cathode side of a sediment microbial fuel cell improved the power generation 1.3 times compared with bare cathode alone. This increase in power generation was attributed to the in situ oxygen produced by the microalgae [253].

To obtain economically viable microalgal-derived biofuels and bioproducts, major advancements in microalgal biology and strain generation, as well as downstream processing, are necessary. Conventional algal research based on mass culture techniques is time-consuming and highly labor-intensive. The microfluidics approach for microalgal production and algal biofuel research has gained much attention in recent times [254]. The microfluidic approach uses microfabricated devices and lab-on-chip systems to assess the culture conditions. It has several features to advance the microalgal bioproduct/biofuel research compared to conventional methods. These microsystems help effectively monitor, control, and manipulate the cell culture at a nanoscale. Microfluidic bioreactors can be used to identify the suitable microalgal species, co-cultures, and environmental conditions suitable for the generation of particular biomass-based products that are commercially viable [255].

Although several studies have been performed on the use of microalgae-bacterial systems for wastewater treatment, there is a need for further research in optimizing parameters for large-scale units. The main challenge in using algal bacterial systems for real scale systems is the stability of the consortia. The stability of the algal-bacterial consortia relies on the communication pattern between the individual species (exchange of metabolites and molecular signals) and the division of labor. Most of the studies regarding the nutrient removal by microalgae-bacterial consortia are limited to laboratory scale studies which may not be representing the real conditions. The competition between the algae and bacteria for nutrients makes their relationship more complex. Very limited studies are available on the association of algal species and bacteria in wastewater treatment. Further investigation is needed to understand the growth conditions such as light intensity and duration of light/dark cycles, availability of nutrients, and the interaction of diversified consortia present in algal-bacterial systems [35]. The dynamics of algal-bacterial systems are largely unexplored, making them difficult to manage. So there is a need for the development of robust models which can simulate the bioprocesses in algal-bacterial systems accurately. Coupling the computational fluid dynamics and biokinetic parameters of the algal-bacterial systems could be used to model the system and implement it in real scale systems. Most of the algal-bacterial systems studied are laboratory scale and were operated for a relatively very short period of time. Therefore, long term studies are required to assess the stability of these systems. The data from the modeling studies can be coupled with smart automation systems. For instance, Casagli et al. [212] have used the data from modeling studies to control the paddle-wheel velocity in an open raceway pond. The key drivers for wastewater treatment using algal-bacterial systems are reducing greenhouse gases emission, improving energy, and nutrient recovery. However, a detailed techno-economic feasibility analysis of these systems is needed to understand the economic feasibility, energy balance, and productivity of the algal-bacterial biomass as a feedstock for biofuel synthesis in order to convince the end-users to adopt these technologies [21]. The algal-bacterial biomass grown on domestic wastewater can be used for resource recovery via biodiesel synthesis, methane generation, or conversion to biohydrogen for reducing the cost of the treatment and ultimately leading to a circular bio-economy. Finally, a clear understanding of the links among the culture conditions, biochemical composition, operational parameters, harvesting, and biomass conversion to fuel is necessary for developing a sustainable wastewater treatment system using algal-bacterial consortia.

## 10. Conclusion

Algal-bacterial systems have a huge potential for wastewater treatment in sustainable way. The factors which affect the algal-bacterial systems are pH, light intensity, light/dark cycle duration, temperature, nutrients, and mixing conditions. Algal-bacterial systems are likely to be more efficient in removing the nutrients and micropollutants than the conventional treatment systems. The increase in the removal rates can be attributed to the possible symbiotic interactions in the algal-bacterial consortium and additional photodegradation due to light. Several models have been developed. However, most of the modeling studies are limited to laboratory studies and employ limited processes related to algae. More emphasis is needed in the understanding of complex algal-bacterial interactions to include in the modeling, and the hydrodynamic aspects of the systems need to be considered. More research is needed to integrate the data from the modeling studies with smart automated control technologies to make reactor operation easier. To implement algal-bacterial systems at field-scale, more emphasis should be given on i) selection of capable algal-bacterial strains, ii) optimization of light illumination as it is the main limiting factor for photosynthesis, iii) modeling the systems in the long run and optimizing the design and operational parameters iv) life cycle analysis and techno-economic feasibility to assess the reliability of these treatment systems. Biomass conversion into bioproducts such as biodiesel, bioelectricity, biohydrogen, and bioethanol require more research. Advanced studies on ecological engineering are needed to understand the algal-bacterial symbiosis, which helps in advancing the algal biorefineries for the production of valuable biofuels and chemicals.
